# Reaction Pathways over ZnZrO_2_‑Based Catalysts and Catalytic Sorbents

**DOI:** 10.1021/acscatal.5c06895

**Published:** 2025-12-31

**Authors:** Laura Proaño, Jordan Wielang, Christopher W. Jones

**Affiliations:** School of Chemical & Biomolecular Engineering, 1372 Georgia Institute of Technology, Atlanta, Georgia 30332, United States

**Keywords:** CO_2_ capture, methanol, catalytic sorbents, DRIFTS-SSITKA, CCUS

## Abstract

Reactive capture and conversion (RCC) is a process intensification approach that integrates CO_2_ capture and hydrogenation within a single unit, removing the CO_2_ purification and storage steps of traditional process flow schemes. This alters the catalytic step from a traditional steady-state (SS) flow process to a transient capture and conversion cycle, which could lead to product distributions distinct from those observed in conventional SS experiments. Such differences are investigated in the combined capture and hydrogenation of carbon dioxide to methanol over a ZnZrO_2_ catalyst and a ZnZrO_2_ + NaNO_3_/Mg_3_AlO*_x_* catalytic sorbent (CS) using fixed-bed kinetic measurements, *in situ* diffuse reflectance infrared Fourier transform spectroscopy (DRIFTS), and steady-state isotopic transient kinetic analysis-DRIFTS (SSITKA-DRIFTS). Under SS conditions, ZnZrO_2_ produced methanol through sequential hydrogenation of HCOO* and CH_3_O* intermediates. On the contrary, CO was attributed primarily to CO_2_ dissociation at oxygen vacancies, as supported by isotopic shifts and measured reaction orders. For the CS, isotopic switching experiments suggested that monodentate carbonate species (CO_3_^2–^, abbreviated as m-CO_3_^2–^) act as active intermediates that can be hydrogenated to HCOO* and subsequently to CH_3_O. Under RCC conditions, *in situ* DRIFTS and isotopic experiments reveal that m-CO_3_^2–^ species formed during the CO_2_ capture step follow two competing routes upon H_2_ exposure: (i) direct hydrogenation to methane on the sorbent domain or (ii) migration of m-CO_3_^2–^ to the ZnZrO_2_ domain, where they are hydrogenated to methanol through the HCOO pathway. Overall, RCC enables carbonate hydrogenation routes not observed under SS cofeed conditions. Thus, the reaction pathways and rates during RCC can be different from operation under conventional SS conditions, and the product distribution is determined here by competition between carbonate hydrogenation on sorbent sites and migration to ZnZrO_2_ for methanol synthesis.

## Graphical abstract

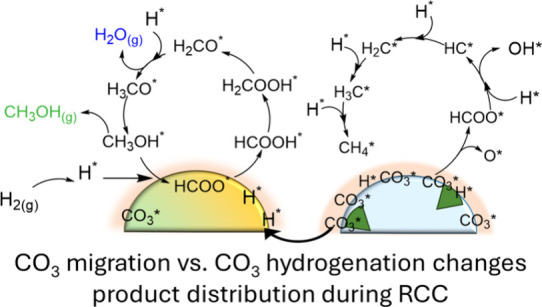

## Introduction

Reactive capture and conversion (RCC) has emerged as a process intensification strategy for conventional carbon capture and utilization (CCU) that could potentially reduce the need for some energy-intensive steps, such as CO_2_ purification, compression, and transport. This has motivated the development of catalytic sorbents (CS) or dual-function materials (DFMs), that integrate a CO_2_ adsorbent with a catalytic functionality. As a result, research efforts have focused on evaluating a wide range of CS by tuning both the CO_2_-adsorbing phase—commonly alkali or alkaline-earth oxides such as Na, Ba, Li, and Ca—and the catalytic domain, including metals like Ni, Ru, and Cu on various supports.^1–5^ These studies have explored various CO_2_ hydrogenation pathways (eqs 1–4), targeting CH_4_, MeOH, or CO as products, the latter via the reverse water–gas shift (RWGS) or dry reforming of methane (DRM).

$${\mathrm{CO}}_{2}+H_{2}↔\mathrm{CO}+H_{2}O\Delta H^{{}^{\circ}}=41.2\;\mathrm{kJ}/\mathrm{mol}\;\Delta G^{{}^{\circ}}=-20.1\;\mathrm{kJ}/\mathrm{mol}$$ 1

$${\mathrm{CO}}_{2}+{\mathrm{CH}}_{4}↔2\mathrm{CO}+2H_{2}\Delta H^{{}^{\circ}}=247.3\;\mathrm{kJ}/\mathrm{mol}\;\Delta G^{{}^{\circ}}=41.7\;\mathrm{kJ}/\mathrm{mol}$$ 2

$${\mathrm{CO}}_{2}+4H_{2}↔{\mathrm{CH}}_{4}+2H_{2}O\Delta H^{{}^{\circ}}=-252.9\;\mathrm{kJ}/\mathrm{mol}\;\Delta G^{{}^{\circ}}=-130.8\;\mathrm{kJ}/\mathrm{mol}$$ 3

$${\mathrm{CO}}_{2}+3H_{2}↔{\mathrm{CH}}_{3}\mathrm{OH}+H_{2}O\Delta H^{{}^{\circ}}=-49.5\;\mathrm{kJ}/\mathrm{mol}\;\Delta G^{{}^{\circ}}=-9.0\;\mathrm{kJ}/\mathrm{mol}$$ 4

Farrauto’s group reported the feasibility of RCC for CH_4_ production in 2015.^6^ Since then, studies probing the impact of catalyst composition, support effects, operating conditions, and impurities (i.e O_2_, SOx) on CH_4_ production have been conducted.^7–11^ In addition to assessing RCC performance, several studies have focused on elucidating the reaction pathways, disentangling the individual contributions of the catalyst and sorbent phase. Proaño et al. and Cimino et al. employed *in situ* DRIFTS during RCC cycles to investigate the formation of surface intermediates, proposing that bidentate carbonates (b-CO_3_*) formed on Na–O sites during CO_2_ capture act as key intermediates in CH_4_ formation over Na_2_O/Ru/Al_2_O_3_ and Li–Na/Ru/Al_2_O_3_ DFMs, respectively.^12,13^ Park et al. studied the reaction mechanism and kinetics under cofeed steady state (SS) conditions for a conventional Ru/Al_2_O_3_ catalyst and a NaNO_3_-promoted Ru/Al_2_O_3_ CS using fixed-bed kinetic measurements and steady-state isotopic transient kinetic analysis (SSITKA)-DRIFTS.^14^ He reported a change in reaction pathways between the catalyst and CS. Specifically, he identified linear carbonyl species as a key reaction intermediate over the catalyst while for the CS, carbonate (CO_3_^2–^), formate (HCOO) and linear carbonyl (C–O) species were identified as potential kinetically relevant intermediates.

Additionally, production of CO and syngas (CO + H_2_) has been explored through RCC via RWGS (eq 1) and DRM (eq 2). Merkouri et al.^15^ used a NiRu/Na_2_O/CeO_2_–Al_2_O_3_ CS for both RWGS and DRM under different operating conditions. Over this material, they identified b-CO_3_* and linear carbonyl species on the surface. For RWGS, the authors suggested that when the feed is switched from CO_2_ to H_2_, preadsorbed linear carbonyl species first desorb from the metal surface. This frees up active sites, allowing H_2_ to dissociatively adsorb. The resulting surface hydrogen then hydrogenates the b-CO_3_* species to CO. In contrast, during DRM, CO is formed by the gasification of carbonaceous deposits formed by decomposition of CH_4_. On the other hand, Hill et al. evaluated a K/Zn_*x*_Al_*y*_O CS for CO_2_ conversion to CO by *in situ* DRIFTS and proposed that adsorbed b-CO_3_* species are hydrogenated to formate (HCOO*) and then CO* results from the decomposition of HCOO*.^16^

Recently, CO_2_ hydrogenation to MeOH has also been explored using Cu/ZnO/Al_2_O_3_ (CZA) and ZnZrO_2_ catalysts doped with K, Ca and Na species (CO_3_^2–^/NO_3_^–^) to make catalytic sorbents. Jeong-Potter et al. explored RCC over a K-impregnated CZA catalyst and reported that CO was produced with the highest selectivity under cofeed SS conditions while MeOH was the main product under cyclic CS sorption/reaction cycles. They associated this shift in product distribution with a possible change in the reaction mechanism because a new CO_3_* band was observed by DRIFTS during CO_2_ adsorption over K/CZA that did not appear over the CZA.^17^

While there are different reaction pathways proposed for transient RCC cycles compared to conventional SS catalysis, there is no direct comparison of the same CS material under both SS and RCC conditions. For example, Park et al.^18^ used Ru/Al_2_O_3_ + NaNO_3_/MgO for RCC but their kinetic studies are for NaNO_3_/Ru/Al_2_O_3_.^14^ Porta et al. reported that alkali impregnation negatively affected CH_4_ selectivity under SS cofeed conditions over a Ru/Al_2_O_3_ catalyst, but their mechanistic studies using *in situ* DRIFTS were conducted only under RCC conditions.^5^ In a similar manner, Cimino et al.^19^ observed the same detrimental impact of alkali addition on CH_4_ selectivity during SS conditions over a Ru/TiO_2_ catalyst and explored the reaction pathways by *in situ* DRIFTS under such SS conditions. Other works by Cimino et al.^12^ exploring CO_2_ methanation pathways under RCC conditions are for a Ru/Al_2_O_3_ catalyst. Finally, Merkouri et al. explored possible reaction pathways for CO_2_ methanation, RWGS and DRM over NiRu/Na_2_O/CeO_2_–Al_2_O_3_ by *in situ* DRIFTS, but only under RCC conditions.^15^ Therefore, a systematic experimental comparison of RCC and cofeed SS conditions over the same materials is needed to better understand how RCC conditions influence the reaction mechanism.

In our previous study, we used a physical mixture of ZnZrO_2_ (ZZO) catalyst and NaNO_3_/Mg_3_AlO*_x_* sorbent as a CS for CO_2_ hydrogenation to MeOH.^20^ In that work, we reported structural characterization of various catalytic sorbent configurations and evaluated the materials under both steady-state (SS) cofeed and reactive capture and conversion (RCC) conditions. We observed a poor correlation between cofeed SS and reactive capture and conversion (RCC) performance, including the unexpected formation of CH_4_ under RCC conditions—absent during SS operation. Additionally, MeOH selectivity increased as the catalyst–sorbent proximity increased, suggesting a synergistic interaction between the catalyst and sorbent domains. However, while these findings highlighted key differences between RCC and SS operation, the mechanistic origins of these differences remain unclear. Specifically, there is uncertainty regarding how surface intermediates and reaction pathways evolve under RCC compared to SS conditions, and whether the CS follows distinct mechanisms under these two modes of operation.

This work probes the mechanisms and kinetics of CO_2_ hydrogenation over a ZZO + 10NaNO_3_/Mg_3_AlO*_x_* catalytic sorbent under both SS cofeed and RCC conditions. We combined fixed-bed kinetic measurements, *in situ* DRIFTS spectroscopy, including DRIFTS-SSITKA, to investigate how surface intermediates, reactivity, and product selectivity evolve under each mode of operation. Comparisons with ZZO (catalyst-only) and 10NaNO_3_/Mg_3_AlO_*x*_ (sorbent-only) reference materials were used to clarify how integrating adsorption and catalytic domains influences reaction pathways, surface intermediates, and overall RCC performance.

## Materials and Methods

### Catalytic Sorbent Preparation

All materials were prepared following the same procedure as reported in our previous work.^20^ ZnZrO_2_ catalyst (ZZO) was synthesized by coprecipitation using Zn­(NO_3_)_2_·6H_2_O and Zr­(NO_3_)_4_·5H_2_O as precursors and (NH_4_)_2_CO_3_ as the precipitating agent, with a Zn:Zr molar ratio of 1:6. The sorbent support (Mg_3_AlO_*x*_) was prepared by calcination of a synthesized Mg–Al–CO_3_ hydrotalcite with a Mg:Al molar ratio of 3 at 400 °C in static air. Finally, the NaNO_3_ promoted sorbent was prepared by impregnating the Mg–Al–CO_3_ with a 2 M NaNO_3_ aqueous solution targeting a final Na wt % loading of 4.6%. Then, the sorbent was calcined at 400 °C in static air. Finally, for the CS, ZnZrO_2_ and Na/Mg_3_AlO_*x*_ were manually ground together in a mass ratio of 6:4 and then the mixture was pelletized, crushed and sieved between 125 and 425 μm. The textural and structural properties of these catalysts, sorbents and catalytic sorbents have been characterized by BET, XRD, CO_2_-TPD, and H_2_-TPR analyses, and the corresponding results were reported in our previous work.^20^

### Steady State Reaction Measurements

Catalytic activity of the ZZO catalyst and ZZO + Na/Mg_3_AlO_*x*_ CS was evaluated under steady-state conditions in a 1/4′ 316 stainless steel fixed-bed reactor housed within 12′ custom aluminum blocks heated by two 550 W Chromalox cartridge heaters. Feed gas flows were controlled via mass flow controllers (Brooks Instruments), and reactor pressure was maintained using a backpressure regulator (TESCOM 26-1700) coupled to an ER3000 electronic pressure regulator. The outlet stream was analyzed using an online 7890 Agilent gas chromatograph (GC) equipped with two TCD and one FID detector.

For all experiments, 0.22 g of pelletized and sieved (125–425 μm) material was placed between SiC beds and reduced *in situ* under H_2_ at 400 °C for 2 h. The reactor was then cooled to the desired temperature and pressurized with the appropriate CO_2_/H_2_/N_2_ gas mixture. Activation energy was measured at 6 bar total pressure using a feed composition of 10% CO_2_, 44% H_2_, balance N_2_, across a temperature range of 260–320 °C. While high pressures (≥20 bar) are typical in steady-state CO_2_-to-MeOH studies, a moderate total pressure was employed in this work to allow comparison between steady-state and RCC hydrogenation results. Reaction orders were determined by varying the CO_2_ partial pressure (0.2–1.7 bar) at fixed H_2_ pressures (2 and 4 bar), and by varying H_2_ partial pressure (0.4–5.1 bar) at fixed CO_2_ pressures (0.6 and 1.6 bar), using N_2_ as balance gas. WHSV was kept at 21200 mL/min/g_cat_. Steady state was confirmed when GC peak areas changed <1% and carbon balance was 100 ± 4%.

### Reactive Capture and Conversion

Reactive capture and conversion (RCC) experiments were carried out in the same setup used for the steady state catalytic activity experiments. After reduction in pure H_2_ at 400 °C 1 h, the reactor was cooled to the desired reaction temperature in N_2_. Then, the capture step was conducted by flowing 10% CO_2_/N_2_ for 30 min and CO_2_ signal was monitored by an IR sensor (Quantek). The CO_2_ uptake was calculated from the breakthrough curves between a blank experiment (reactor filled with only SiC, keeping total volume bed constant) and the CO_2_ capture step. Next, the feed was switched to N_2_ and the reactor was purged for 10 min, until the CO_2_ reading was below 0.5%. Finally, for the conversion step, the feed was switched to H_2,_ and the reactor was pressurized to 6 bar. Different H_2_ concentrations (30%, 70% and 100%) were evaluated to gain understanding of H_2_ effect on the RCC kinetics and product distribution. Once the reactor reached the set pressure, the effluent was analyzed using the GC and the conversion step was run until no more products were detected. The total RCC productivity and rate were then calculated by integrating the area under the flow vs time curves for each product.

### *In Situ* DRIFTS Experiments

*In situ* DRIFTS experiments were performed in a BRUKER Invenio spectrometer equipped with a Harrick high-temperature DRIFTS cell and ZnSe windows. Spectra were collected at a resolution of 4 cm^–1^ with 60 scans per spectrum. A total gas flow of 100 mL/min was maintained in all experiments, and flows were controlled using a Bronkhorst mass flow controller.

Samples (ZZO, ZZO + 10NaNO_3_/Mg_3_AlO_*x*_, or 10NaNO_3_/Mg_3_AlO_*x*_) were first reduced *in situ* under pure H_2_ at 400 °C and 6 bar for 2 h. After reduction, the cell was purged with Ar, cooled to the reaction temperature, and a background spectrum was recorded. For cofeed SS conditions, the sample was exposed to a gas mixture with a 10% CO_2_/40% H_2_ balance Ar at 6 bar. Pressure was controlled using a Swagelok spring-loaded backpressure regulator. Once steady state was achieved, the feed was switched to H_2_ at the same pressure, and spectra were collected to monitor the adsorbed species.

For RCC conditions, after reduction, the sample was cooled in Ar to the reaction temperature and a background spectrum was recorded. A 30 min CO_2_ adsorption step was then performed under 10% CO_2_/Ar at atmospheric pressure, this was followed by a 5 min Ar purge. Finally, the feed was switch to H_2_ and the pressure was raised to 6 bar.

### DRIFTS-SSITKA

DRIFTS-SSITKA experiments were performed using the same BRUKER Invenio spectrometer and Harrick high-temperature DRIFTS cell described in Section 2.4. A 3-way valve was used to alternate between ^12^CO_2_ and ^13^CO_2_ feeds during experiments, while maintaining a total gas flow of 100 mL/min. Gas-phase signals were monitored downstream using a Pfeiffer mass spectrometer, tracking *m*/*z* signals for ^12,13^CO_2_ (*m*/*z* = 44,45), ^12,13^CO (*m*/*z* = 28,29), ^12,13^CH_4_ (*m*/*z* = 16,17) and ^12,13^MeOH (*m*/*z* = 31,32,33). For SS cofeed conditions, after *in situ* reduction in H_2_, the sample was exposed to a ^12^CO_2_/H_2_ mixture (CO_2_:H_2_ = 1:4) until steady state was reached—confirmed by stable IR and mass spectrometry signals. The feed was then switched to a ^13^CO_2_/H_2_ mixture using the 3-way valve, maintaining constant flow and pressure. DRIFTS spectra and mass spectrometry signals were collected throughout the isotope switch to track gas-phase and surface species evolution.

## Results and Discussion

### CO_2_ Adsorption Mechanism on 10NaNO_3_/Mg_3_AlO*_x_*

Figure 1A presents *in situ* DRIFTS spectra of the 10NaNO_3_/Mg_3_AlO_*x*_ sorbent during activation (heating up in Ar to 400 °C and after reduction in H_2_). At room temperature, different bands are observed, with Table 1 presenting a summary of the assigned bands. With increasing temperature, we observe the evolution of different bands. The O–H signal at 3627 cm^–1^ decreases in intensity, consistent with loss of H_2_O. As temperature increases, NO_3_^–^ (835, 1383, 1789, 2091, 2493, 2769, 2849, 2921 cm^–1^) related bands decrease in intensity, while the NO_2_^–^ bands (1051, 1268, 1762, 2072, 2767 cm^–1^) increase, indicating the decomposition of NaNO_3_ (eq 5).^21–23^ The melting points of NaNO_3_ and NaNO_2_ are 310 and 270 °C, respectively. Thus, under the sorption conditions NaNO_3_/NaNO_2_ is present in a molten state containing [Na^+^···NO_3_^–^] and [Na^+^···NO_2_^–^] ionic pairs.^24,25^ Additional bands at 1656 and 1530 cm^–1^, corresponding to ν­(HOH) and ν­(OCO) vibrations of interlayer H_2_O and CO_3_^2–^, respectively, are also observed. Upon heating, these bands disappear from the spectra between 200 and 300 °C.^25–28^

$${\mathrm{NaNO}}_{3}↔[{\mathrm{Na}}^{+}\cdot \cdot \cdot {\mathrm{NO}}_{-}^{2}{]}_{\mathrm{melt}}+\frac{1}{2}O_{2}$$ 5

**Figure 1:**
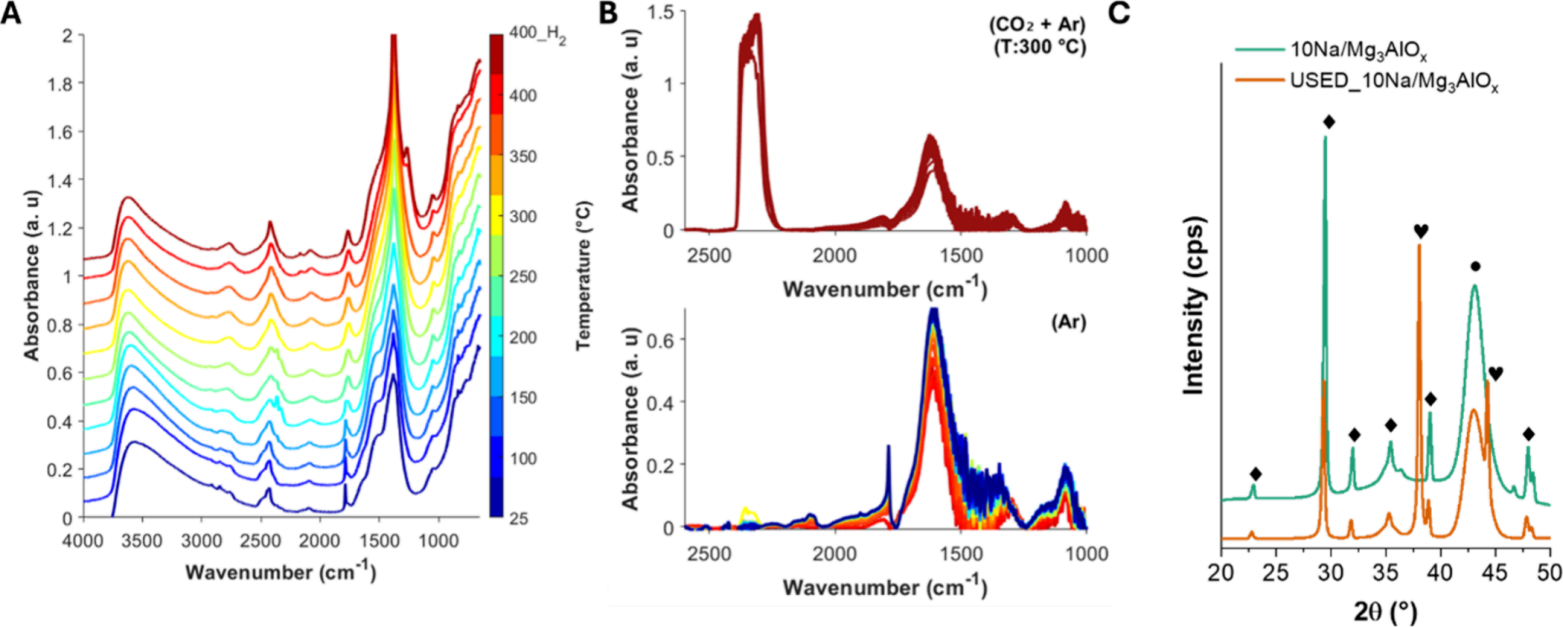
*In situ* DRIFTS spectra. (A) 10NaNO_3_/Mg_3_AlO*_x_* during activation heating up in Ar to 400 °C and then after reduction in H_2_. (B) During CO_2_ exposure at 300 °C followed by cooling down to RT in Ar. (C) XRD pattern of fresh and CO_2_ exposed 10NaNO_3_/Mg_3_AlO*_x_*. (Diamond) NaNO_3_, (circle) MgO, and (heart) Na_2_MgCO_3_.

**Table 1:** IR Band Assignments

species	IR band	assignment^a^	reference
NO^3–^	1383	ν_sym_(NO)	^21–23^
	835	π(NO)	
	1789	ν_sym_(NO) + δ(NO)	
	2091	ν_sym_(NO) + ν_asym_(NO)	
	2493	ν_asym_(NO) + π(NO)	
	2769	2(ν_asym_)	
	2849	ν_asym_(NO) + 2π(NO)	
	2921	ν_asym_(NO) + δ(NO) + ν_sym_(NO)	
NO^2–^	1051	π(NO)	^21–23^
	1268	δ(NO)	
	1762	ν_sym_(NO) + δ(NO)	
	2072	ν_sym_(NO) + ν_asym_(NO)	
	2767	2(ν_asym_)	
H_2_O/OH	3627	ν(OH)	^26–28^
	1640	ν(HOH)	^26^
CO_3_^2–^ interlayer	1530	ν(OCO)	^26–28^
m-CO_3_^2–^	1601	ν_asym_(OCO)	^27,29–32^
	1080	ν_sym_(OCO)	
HCOO	1373	ν_sym_(OCO)	^35–37^
	1586	ν_asym_(OCO)	
	2736	δ(CH) + ν_sym_(OCO)	
	2965	δ(CH) + ν_asym_(OCO)	
	2875	ν(CH)	
CH_3_O	1051	ν(CO)-terminal	^35–37^
	1140	ν(CO)-bridge	
	2827	ν_sym_(CH_3_)	
	2928	ν_asym_(CH_3_)	
CH_4_	3016	ν_sym(_CH)	^14^

a Totally symmetric stretching (ν_sym_), asymmetric stretching (ν_asym_), out of plane bending (π), in-plane bending (δ), and overtones (2­(ν_i_)).

Figure 1B presents *in situ* IR spectra of the NaNO_3_/Mg_3_AlO_*x*_ sample between 2500 and 1000 cm^–1^ at 300 °C under CO_2_ flow. Under these conditions, peaks at 1080 cm^–1^ and 1600 cm^–1^ appear, corresponding to ν_sym_(OCO) and ν_asym_(OCO) of monodentate carbonate (m-CO_3_^2–^).^27,29–32^ Upon switching to Ar and cooling to RT, the NO_3_ peak at 1787 cm^–1^ reappears. This suggests that NaNO_3_ is not consumed during the CO_2_ adsorption, and that the observed m-CO_3_^2–^ band is likely associated with carbonate formation on the MgO domain. However, XRD of CO_2_ exposed NaNO_3_/Mg_3_AlO_*x*_ (Figure 1C) has two new Bragg reflections at 38° and 44°, which are likely associated with formation of a Na_2_Mg­(CO_3_)_2_ phase. Previous reports of CO_2_ adsorption on NaNO_3_/MgO sorbents have also reported the formation of Na_2_Mg­(CO_3_)_2_ at high temperature.^25,33^ We note that although we use adsorption throughout the paper for consistency with prior RCC and CO_2_-capture literature, both adsorption through surface interactions and absorption leading to bulk-phase carbonates occur during the CO_2_ capture step in this work.

Gao et al.^29^ and Rekhtina et al.^25^ studied CO_2_ adsorption on NaNO_3_/MgO sorbents by *in situ* time-resolved XRD experiments, isotopic labeling experiments and DFT calculations. These studies proposed that at high temperature, NaNO_3_ partially decomposes to NaNO_2_ and is present in a molten state. Additionally, Rekhtina et al.^25^ concluded from *in situ* XRD experiments, that at regeneration temperatures ≥450 °C, partial decomposition of NaNO_3_ to NaNO_2_ occurs. Upon carbonation, this leads to the formation of Na_2_Mg­(CO_3_)_2_, which acts as nucleation seeds for further formation of MgCO_3_. For the NaNO_3_/Mg_3_AlO_*x*_ sorbent, NO_3_ decomposition is also observed (Figure 1A), even at the lower reduction temperature of 400 °C, likely because of the reductive H_2_ atmosphere that can promote NO_3_-to-NO_2_ decomposition at temperatures below those reported under N_2_. Based on DFT calculations, Gao et al.,^29^ and Rekhtina et al.^25^ proposed that molten NO_3_/NO_2_ could weaken the Mg–O bond, resulting in dissolution of [Mg^2+^···O^2–^] ionic pairs into the molten media. Thus, the authors proposed that CO_2_ diffuses in the molten media and upon interaction with the [Mg^2+^···O^2–^] ionic pairs it forms CO_3_^2–^. After reaching saturation, Na_2_Mg­(CO_3_)_2_ or MgCO_3_ precipitates (Scheme 1).^34^ For Mg–Al mixed metal oxides with a high Mg/Al ratio, upon calcination, only MgO diffraction peaks are observed at 42° and 62° 2θ (Figure 1C).^28^ Accordingly, it is generally accepted that these materials follow the same CO_2_ adsorption mechanism as NaNO_3_/MgO. However, the presence of Al in the Mg_3_AlO_*x*_ sorbent, may increase the dispersion of NaNO_3_, resulting in more Na–Mg interfacial sites, favoring precipitation of Na_2_Mg­(CO_3_)_2_ over MgCO_3_. These differences in support composition and activation atmosphere may therefore contribute to the exclusive observation of crystalline Na_2_Mg­(CO_3_)_2_ in this work compared to only-MgO sorbents. Nevertheless, the DRIFTS and XRD data from the CO_2_ capture step suggest that the overall molten-mediated CO_2_ uptake mechanism remains consistent with that previously reported for NaNO_3_/MgO sorbents.

**Scheme 1:**
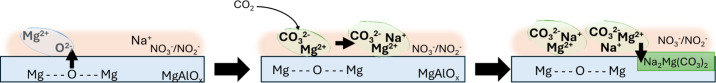
Proposed CO_2_ Adsorption Mechanism over 10NaNO_3_/Mg_3_AlO*_x_*. Adapted from Park, S. J.; Kim, Y.; Jones, C. W. *ChemSusChem.* **2020**, *13*, 2988–2995. Copyright 2020 Chemistry Europe

### MeOH and CO Formation Mechanisms under SS Cofeed Conditions

Figure 2A presents Arrhenius plots for CO and MeOH measured between 260 and 320 °C under conventional SS catalysis conditions using the ZZO catalyst. The apparent activation energies (*E*_app_) estimated from linear regression were 70 and 174 kJ/mol for MeOH and CO, respectively. These values are within the range observed for *E*_app_ of other catalysts reported in the literature. For example, Vergara et al. reported *E*_app_s of 66 and 91 kJ/mol for MeOH and CO, respectively, at 30 bar over a CuCeOx/TiO_2_ catalyst.^38^ Another study by Ding et al. reported *E*_app_ of 81 and 125 kJ/mol for MeOH and CO, respectively, over a ZnZrO_2_ catalyst with a Zn:Zr ratio of 0.25.^35^ Figure 2B presents the dependence of the MeOH and CO formation rates on the CO_2_ partial pressure. MeOH shows a Langmuir-type dependence with respect to CO_2_, consistent with increasing surface coverage and eventual site saturation at higher CO_2_ concentrations, indicating equilibrium of surface species with gas phase CO_2_. On the contrary, CO formation exhibits a first-order dependence on CO_2_ pressure. For the apparent reaction order of H_2_ (Figure 2C), MeOH formation exhibits first order dependence while CO formation shows zero-order dependence. This suggests that CO and MeOH formation may involve different rate-determining steps (RDS). For MeOH synthesis, the RDS likely involves an H-rich intermediate, like HCOOH, or H_2_COOH.^39–42^ In contrast, the near-zero order dependence of CO on H_2_ pressure, combined with its first-order dependence on CO_2_ pressure, suggests a RDS that involves a less hydrogenated intermediate than MeOH, such as direct CO_2_ dissociation or decomposition of formate (HCOO*) or carboxyl (COOH*) intermediates. To further investigate the nature of the surface intermediates responsible for these distinct kinetic behaviors, *in situ* DRIFTS experiments were performed under SS cofeed conditions (Figure 3).

**Figure 2:**
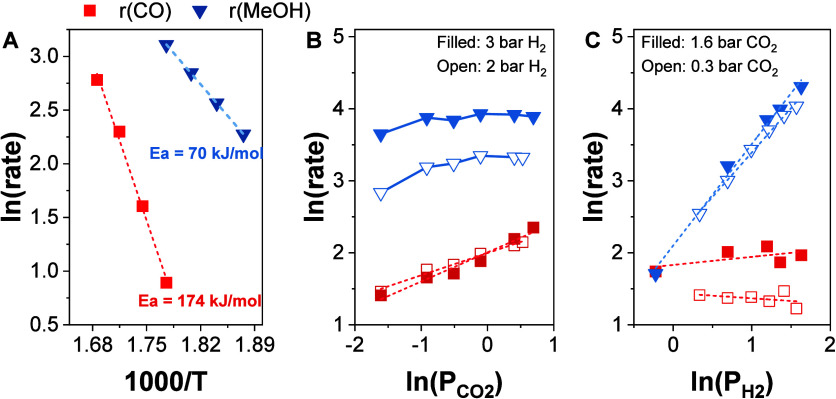
Kinetic measurements over ZnZrO_2_. (A) Arrhenius plots used to calculate the apparent activation energies (*E*_app_) for MeOH (blue-triangles) and CO (red-squares); (B) ln­(MeOH) and ln­(CO) formation rates as a function of ln­(CO_2_) partial pressure; (C) ln­(MeOH) and ln­(CO) formation rates as a function of ln­(H_2_) partial pressure. (*X*_CO2_ < 3%). Total operating pressure was 6 bar and WHSV = 21,200 mL/g_cat_/h.

**Figure 3:**
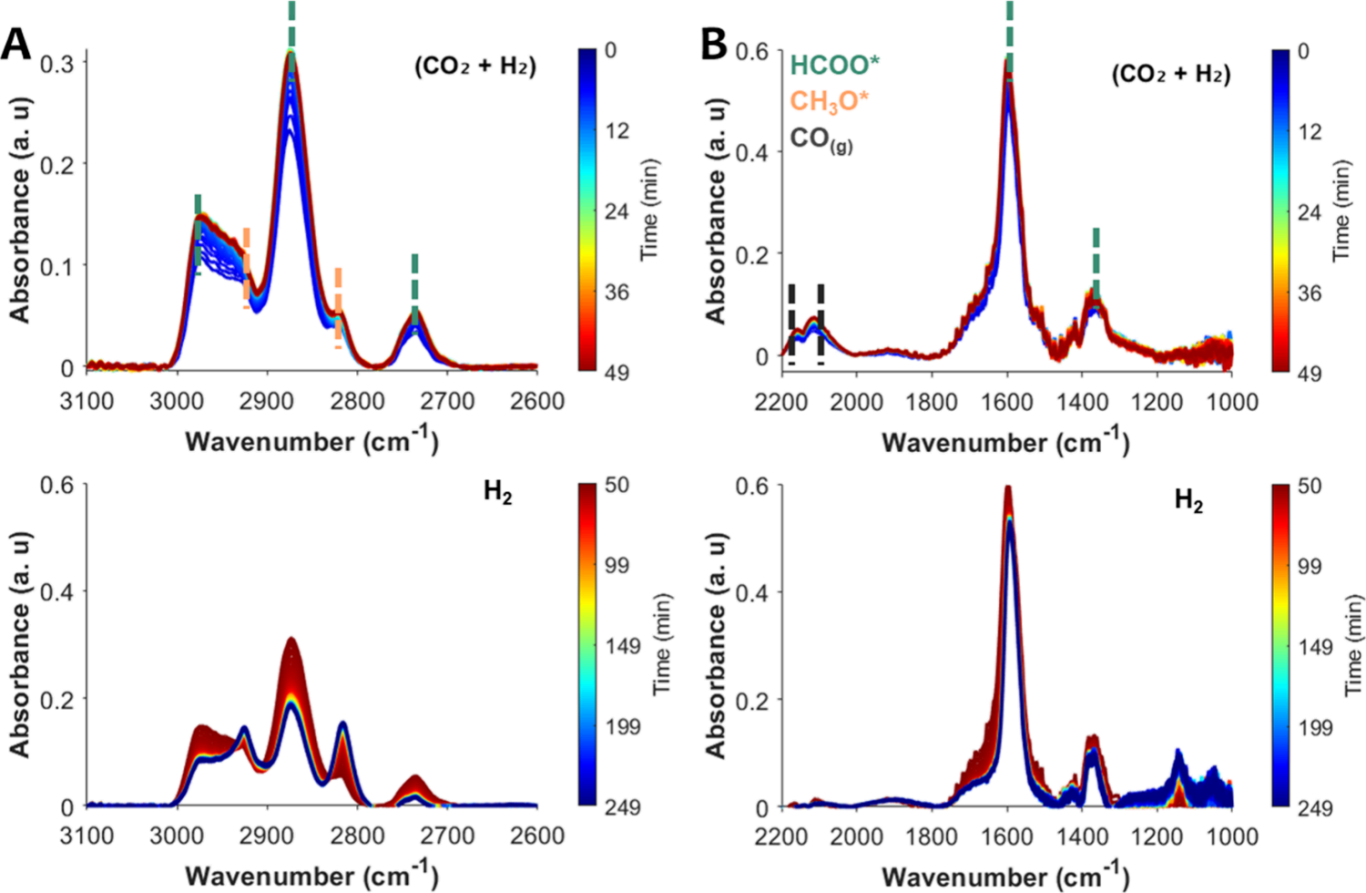
*In situ* DRIFTS over the ZZO catalyst at 6 bar and 300 °C. (A) Spectral region 2600–3100 cm^–1^ and (B) region 1000–2200 cm^–1^. Time evolution of spectra under cofeed SS conditions (10% CO_2_/30% H_2_/Ar, top panel) and after switching the feed to H_2_ (bottom panel).

During SS cofeed conditions (Figure 3A, top panel), characteristic peaks for HCOO* appear at 1371 and 1598 cm^–1^ and are assigned to ν_sym_(OCO) and ν_asym_(OCO) vibrations, alongside peaks at 2736, 2875, and 2965 cm^–1^, assigned to combination peaks of δ­(CH) + ν_aym_(OCO), δ­(CH) and δ­(CH) + ν_asym_(OCO), respectively. Characteristic methoxy (CH_3_O*) peaks also appear as shoulders at 2819 and 2928 cm^–1^, which are assigned to ν_sym_(CH) and ν_asym_(CH) vibrations. Additionally, roto-vibrational modes of gas-phase CO are also observed at 2111 and 2153 cm^–1^.^35,37,38,42,43^ Moreover, a small sharp peak at 2077 cm^–1^ is also observed (Figure S2), which could be assigned to linearly bound C–O species. For metallic surfaces (e.g., Pt or Pd) peaks between 2000 and 2100 cm^–1^ are typically assigned to linearly bound C=O.^44^ In the case of ZnZrO_2_, after reduction in H_2_, this peak could be assigned to C=O adsorbed on under-coordinated Zn^0^ or Zr^3+^ cations. Another possibility is the formation of ketenic-like species on oxygen vacancies, as previously reported for MgO, which exhibit a band at 2086 cm^–1^ during CO adsorption.^23,45^ A similar sharp peak at about 2077 cm^–1^ is observed in the DRIFTS spectra reported in other studies of Zn-ZrO_2_ catalysts. However, the authors of those studies did not discuss possible assignments for the peak.^25,37^ Thus, precise assignment of this C=O feature requires complementary CO adsorption experiments and DFT calculations.

After SS cofeed experiments, the gas flow was switched to H_2_ at the same temperature. Once the CO_2_ feed stops, the CO­(g), and HCOO* bands decreased in intensity and the CH_3_O* peaks increased in intensity (Figure 3). The evolution of CH_3_O* bands closely follows the decay of HCOO* bands, indicating that CH_3_O* likely forms through stepwise hydrogenation of HCOO*. This mechanism has been previously reported as more favorable over ZZO solid solutions as opposed to the RWGS + CO hydrogenation mechanism, supported by DFT calculations.^35,37,42,46^ In contrast, the CO­(g) bands drop sharply upon CO_2_ removal. This suggests that CO is formed primarily via direct CO_2_ dissociation, rather than through HCOO/COOH* decomposition. Still, the decay of HCOO* may reflect both its hydrogenation to CH_3_O* and partial decomposition to CO. Thus, to further investigate the role and reactivity of HCOO* species, DRIFTS-SSITKA experiments were performed by switching from ^12^CO_2_ to ^13^CO_2_ feeds under SS cofeed conditions (Figure 4).

**Figure 4:**
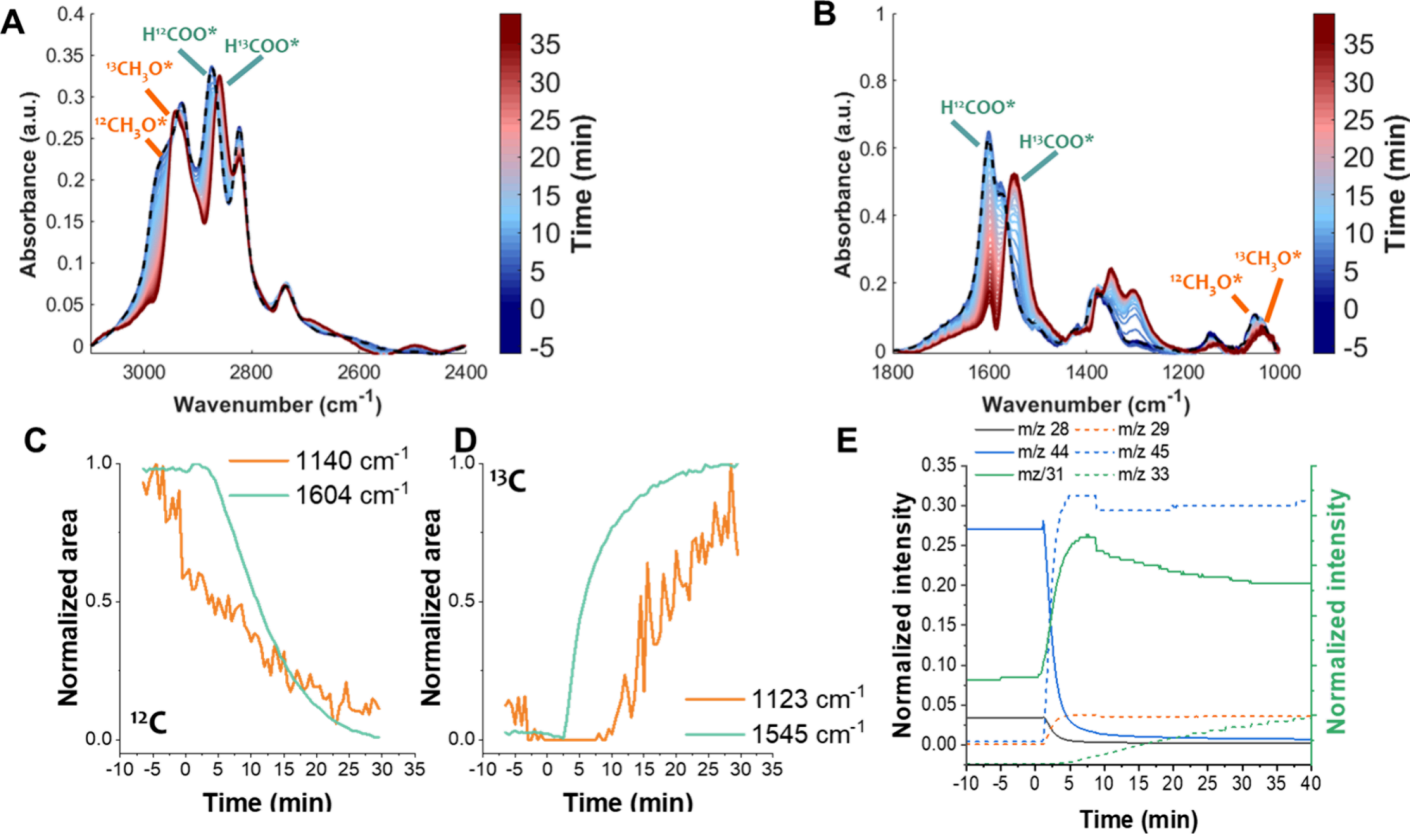
Evolution of *in situ* DRIFTS spectra in the regions (A) 2400–3100 cm^–1^ and (B) 1000–1800 cm^–1^. (C, D) Normalized areas of ^12^CO_2_- and ^13^CO_2_-derived HCOO and C–O components and (E) normalized *m*/*z* intensities detected at the outlet of the reaction cell during ^12^CO_2_ → ^13^CO_2_ switching under cofeed steady-state conditions using the sorbent-free catalyst, ZZO. *t* = 0 indicates the feed switch (^12^CO_2_ → ^13^CO_2_).

Figure 4A,B presents the *in situ* DRIFTS spectra during the ^12^CO_2_/^13^CO_2_ switching in the regions between 1000 and 1800 cm^–1^ and 2400–3100 cm^–1^. It can be observed that fundamental ^12^HCOO peaks at 1376, 1604, and 2964 cm^–1^ shifted to 1352, 1545, and 2943 cm^–1^, respectively. Similarly, the CH_3_O* band related to ν­(CO) at 1140 cm^–1^ shifted to 1120 cm^–1^. Figure 4C,D presents the time evolution of the normalized areas of the ^12/13^HCOO and ^12/13^CH_3_O components during the isotopic switch. The HCOO* bands exhibited a faster transition than the CH_3_O* bands, suggesting that CH_3_O* is formed in a subsequent step derived from the HCOO* intermediate. Figure 4E presents the time evolution of the normalized *m*/*z* signals of ^12^CO_2_/^12^CO (44, 28), ^13^CO_2_/^13^CO (45,29) and ^12^MeOH/^13^MeOH (31 and 33) recorded at the outlet of the DRIFTS cell. The CO_2_ isotope signals switched rapidly, consistent with direct gas-phase replacement. The ^12^MeOH signal displayed a sharp initial increase followed by a decay. A blank experiment revealed that *m*/*z* 31 intensity can be influenced by contributions from *m*/*z* 45 (Figure S5), which may explain the rapid initial rise. However, a subsequent decay in ^12^MeOH intensity is observed, which closely parallels the increase in ^13^MeOH, and both trends align with the replacement dynamics of the ^12/13^CH_3_O* intermediates observed in the IR spectra. Thus, it is suggested that MeOH formation over the ZZO catalyst could occur via a mechanism in which HCOO* and CH_3_O* participate as intermediates (Scheme 2).

**Scheme 2:**
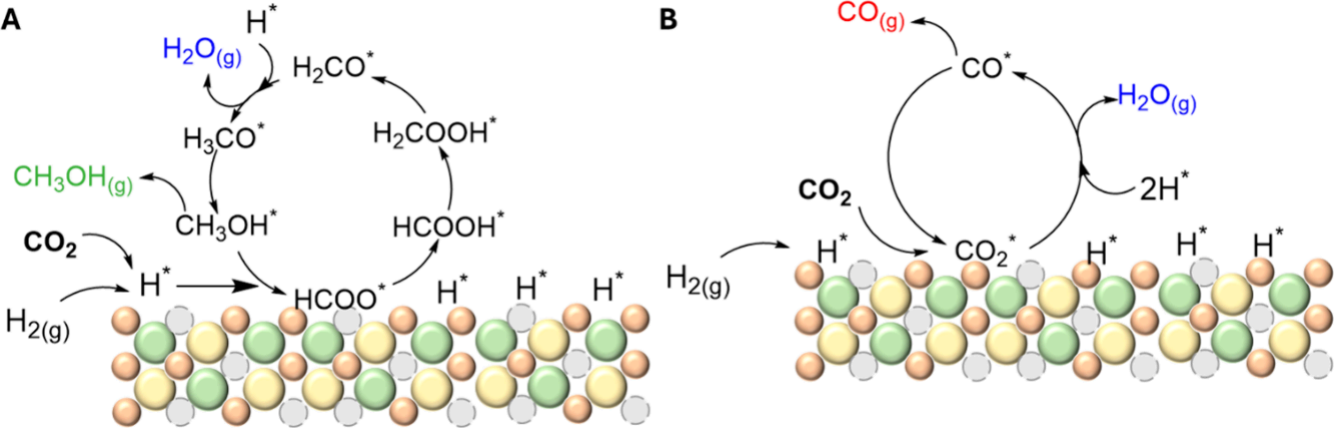
Proposed Reaction Pathways over the ZZO Catalyst: (A) MeOH and (B) CO Mechanisms

For CO formation, the sharp peak at 2077 cm^–1^ exhibits an isotopic shift to 2037 cm^–1^ upon switching between ^12^CO_2_ and ^13^CO_2_ (Figure S6). The sharp exchange between ^12^CO and ^13^CO species (Figure 4C,D) upon switching from ^12^CO_2_ to ^13^CO_2_ indicates that CO formation closely follows the gas-phase replacement of CO_2_, as adsorbed CO_2_ dissociates on Zn^0/+1^ or Zr^3+^ sites near oxygen vacancies, and the O atom fills the vacancy, thus reoxidizing the cation and evidencing a redox RWGS mechanism. Previous reports have shown by EPR studies that H_2_ pretreatment of ZZO generates asymmetric oxygen vacancies (i.e., Zn–O_v_–Zr), which could act as redox sites, and may provide the site for the redox RWGS mechanism.^42,47,48^ Fixed-bed kinetics revealed a first-order dependence on CO_2_ for CO formation, consistent with a mechanism involving CO_2_ dissociation (Figure 2). DRIFTS spectra in other studies also show a sharp feature near ∼2077 cm^–1^, yet this is not discussed in detail.^25,42^ Additionally, the DFT calculations in those works suggest that the COOH/HCOO decomposition pathway is more energetically favored.^25,42^ Thus, it is possible that the observed C=O peak arises from this pathway. Nevertheless, in the current work, the isotopic exchange of the 2077/2037 cm^–1^ band occurs more rapidly than the HCOO transition (Figure 4C,D). Thus, both pathways could contribute to CO formation on ZZO, and contributions from the redox mechanism may have been previously overlooked.

For CO_2_ hydrogenation over the CS, ZZO + Na/Mg_3_AlO_*x*_, a change in the *E*_app_ was observed compared to the catalyst, ZZO, alone, which could suggest a change in the RDS. For MeOH, an *E*_app_ of 84 kJ/mol was estimated, whereas for CO it was 155 kJ/mol. Figure 5B presents the dependence of the MeOH and CO formation rates on the CO_2_ partial pressure, where it can be observed that MeOH formation had a negative dependence on the CO_2_ partial pressure (i.e., negative CO_2_ reaction order). This is expected since the inclusion of the NaNO_3_/Mg_3_AlO_*x*_ increases the amount of CO_2_ on the surface. The decrease in reaction order relative to the pure catalyst, ZZO, was also observed for CO, changing from first order to zero order. Considering a classical Langmuir dependence, the negative reaction order is consistent with a significant adsorption term in the denominator of the rate law. However, this alone does not indicate whether the additional adsorbed CO_2_ species are active intermediates or just spectators. For the H_2_ dependence (Figure 5C), the MeOH formation rate continues to exhibit a first-order dependence, as for the ZZO catalyst. However, the CO dependence on the H_2_ partial pressure became slightly positive at a fixed CO_2_ partial pressure of 0.6 bar, with no CO detected at low H_2_ partial pressures (<3 bar). When the CO_2_ partial pressure was increased to 1.6 bar, the CO formation rate shifted to an approximately first-order dependence on H_2_. This suggests the involvement of H_2_ in the CO formation pathway, in contrast to the ZZO catalyst, where CO formation was zero order in H_2_ (Figure 2). These changes in apparent activation energy and reaction order suggest a shift in the reaction pathways between the ZZO catalyst and the ZZO + 10NaNO_3_/Mg_3_AlO_*x*_ CS.

**Figure 5:**
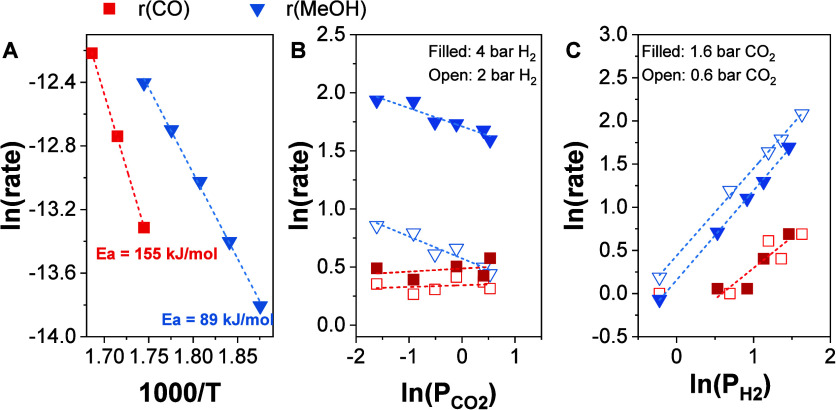
Kinetic measurements over ZnZrO_2_ + 10NaNO_3_/Mg_3_AlO_*x*_. (A) Arrhenius plots used to calculate the apparent activation energies (*E*_app_) for MeOH (blue-triangles) and CO (red-squares); (B) ln­(MeOH) and ln­(CO) formation rates as a function of ln­(CO_2_) partial pressure (*X*_CO2_ < 3%); (C) ln­(MeOH) and ln­(CO) formation rates as a function of ln­(H_2_) partial pressure. Total operating pressure was 6 bar and WHSV = 21,200 mL/g_cat_/h.

Compared to the ZZO catalyst, where only HCOO*/CH_3_O* bands were detected (Figure 3), the DRIFTS spectra of ZZO + 10NaNO_3_/Mg_3_AlO_*x*_ under SS cofeed conditions exhibit an intense band at about 1600 cm^–1^ and a small band at 1081 cm^–1^ (Figure 6), assigned to the ν_asym_(OCO) and ν_sym_(OCO) of m-CO_3_^2–^. In parallel, the HCOO* bands at 2736, 2852, and 2965 cm^–1^ are still observed, alongside CH_3_O*-related shoulders at 2814 and 2936 cm^–1^, similar to those observed over the ZZO catalyst. Thus, over the CS, CO_2_ is activated over both the ZZO domain and the NaNO_3_/Mg_3_AlO_*x*_ domain (vide infra). Additionally, the vibrational bands of gas phase CO between 2100 and 2200 cm^–1^ appeared in the spectra. Upon switching to H_2_, the m-CO_3_^2–^ bands at 1601 cm^–1^ slightly decreased in intensity, while the CH_3_O* bands slightly increased. Moreover, the peak at 3016 cm^–1^, which is a C–H vibration related to CH_4_, increased in intensity under the high partial pressure of H_2_.^14^ The CO­(g) band intensity sharply decreased once the CO_2_ feed stopped, also suggesting a direct CO_2_ dissociation mechanism, as observed for the ZZO catalyst.

**Figure 6:**
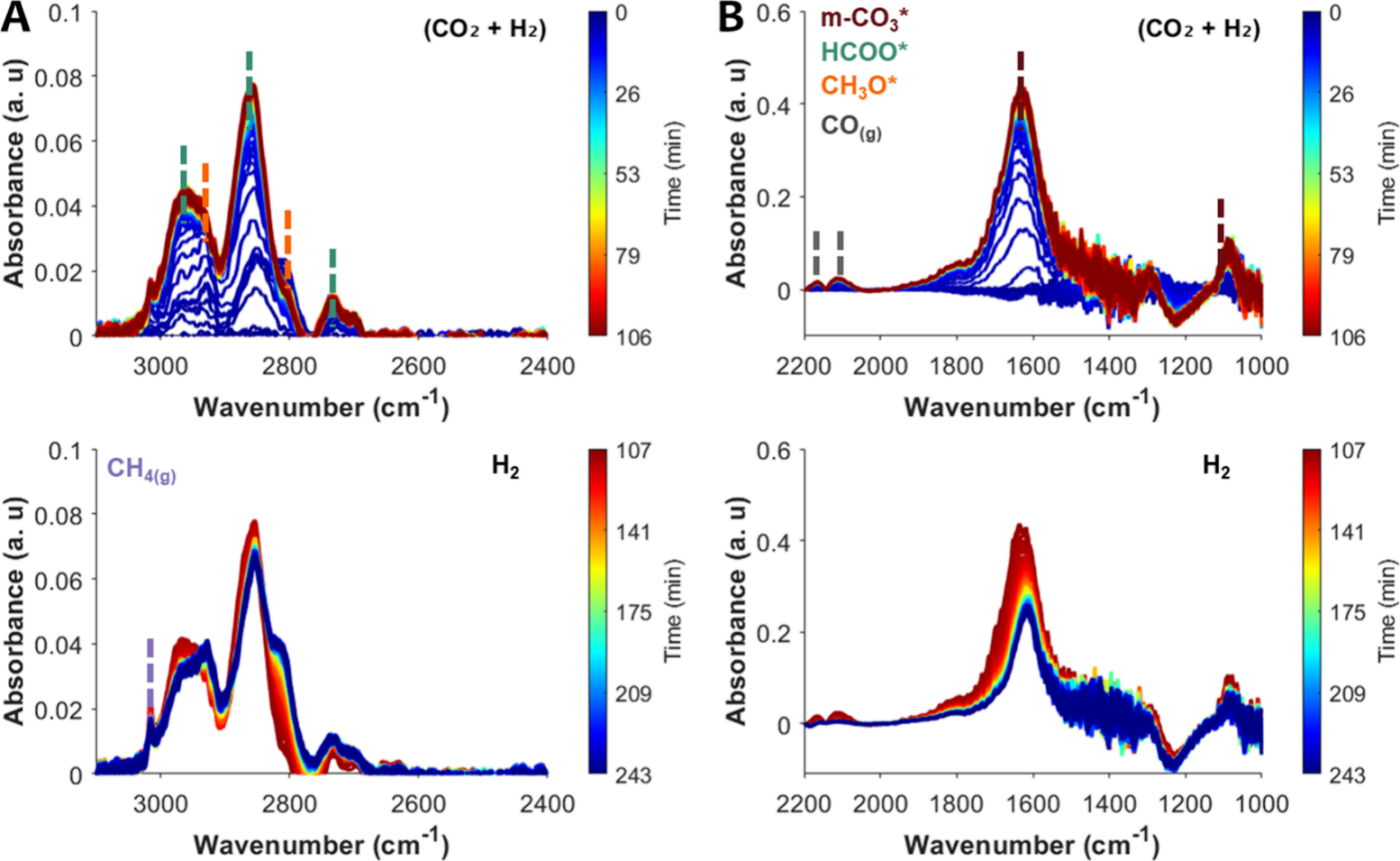
*In situ* DRIFTS under SS cofeed conditions using the CS, ZZO + 10NaNO_3_/Mg_3_AlO*_x_*. (A) Spectral region 2600–3100 cm^–1^ and (B) region 1000–2200 cm^–1^. 10% CO_2_/30% H_2_/Ar (top panel) and after switching the feed to H_2_ (bottom panel).

During SSITKA-DRIFTS experiments (Figure 7) the m-^12^CO_3_^2–^ band at ∼1600 cm^–1^ decreased in intensity as the m-^13^CO_3_^2–^ band at ∼1535 cm^–1^ appeared. Similarly, ^12^HCOO and ^12^CH_3_O* bands between 2800 and 2900 cm^–1^ decreased in intensity, while ^13^HCOO and ^13^CH_3_O* species increased in intensity, indicating isotopic replacement (Figure 7A,B). The decrease in the m-^12^CO_3_^2–^ (∼1600 cm^–1^) band aligns with the decay of the ^12^HCOO (2852 cm^–1^), while the increase in the m-^13^CO_3_^2–^ band aligns with the appearance in the ^13^HCOO (1535 and 2844 cm^–1^, respectively). Meanwhile, the decrease and increase of the ^12^CH_3_O* (2936 cm^–1^) and ^13^CH_3_O* (2926 cm^–1^) bands occurred with a delay relative to the m-CO_3_^2–^ and HCOO transitions. This behavior suggests that CH_3_O forms in a subsequent step from HCOO intermediates. Consistently, an increase in the *m*/*z* 33 signal corresponding to ^13^C MeOH was also detected at the outlet of the DRIFTS cell upon isotopic switching (Figure 7D). These results suggest that m-CO_3_ species can spill over from the sorbent to the ZZO domain, where they undergo hydrogenation to HCOO and then to CH_3_O, ultimately leading to MeOH formation. This spillover may occur through the molten NaNO_3_, which provides a medium for CO_3_^2–^ transfer across the sorbent–catalyst interface.

**Figure 7:**
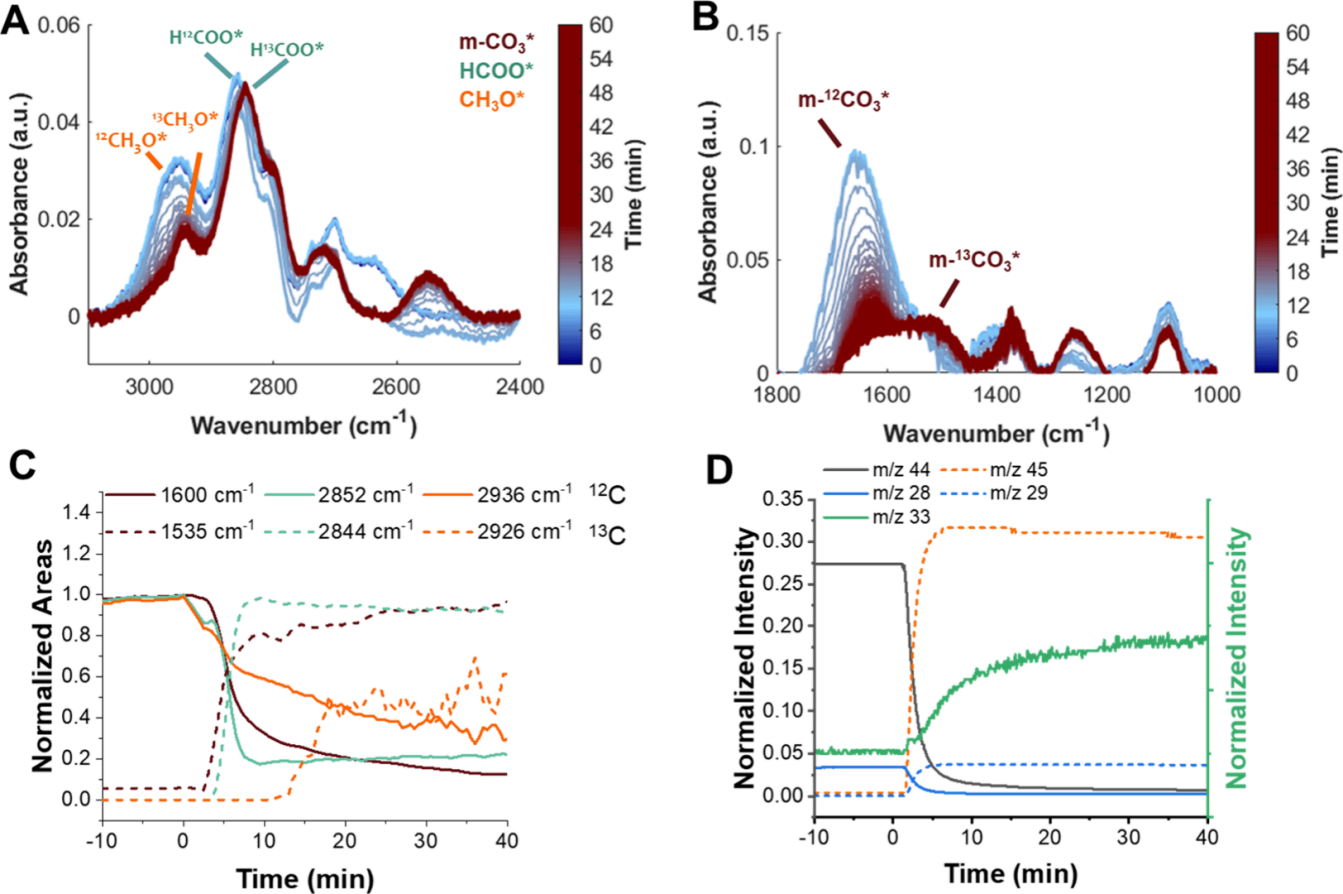
Evolution of *in situ* DRIFTS spectra in the regions (A) 2400–3100 cm^–1^ and (B) 1000–1800 cm^–1^. (C) Normalized areas of ^12^CO_2_- and ^13^CO_2_-derived m-CO_3_*/HCOO*/CH_3_O*. (D) Normalized *m*/*z* intensities detected at the outlet of the reaction cell during ^12^CO_2_ → ^13^CO_2_ switching under SS cofeed conditions over ZZO + 10NaNO_3_/Mg_3_AlO_*x*_. *t* = 0 indicates the feed switch (^12^CO_2_ → ^13^CO_2_).

### Kinetics and Reaction Mechanism under RCC Conditions

In this section, the kinetic behavior of CO_2_ hydrogenation under RCC conditions is examined. Fixed-bed RCC experiments were conducted only for the ZZO + 10NaNO_3_/Mg_3_AlO*_x_* CS (Figure 7), as the ZZO catalyst alone has low CO_2_ uptake (0.3 mmol/g).^20^ Consequently, product signals at lower H_2_ concentrations were too weak to enable accurate quantification on the sorbent-free catalyst. As such, mechanistic insights for the ZZO catalyst under RCC are derived exclusively from *in situ* DRIFTS experiments.

Figure 8 presents the *in situ* DRIFTS spectra during the CO_2_ capture and conversion steps over the ZZO catalyst. During the CO_2_ capture step, a broad band appeared between 1800 and 1200 cm^–1^. Upon peak deconvolution, bands at 1240, 1333, 1401, 1461, 1539, and 1752 cm^–1^ could be assigned to m-CO_3_^2–^, bidentate carbonates, and bicarbonate species (HCO_3_*).^35^ High wavenumber components of HCOO* were also observed at 2736, 2875, and 2965 cm^–1^, indicating adsorption of CO_2_ as HCOO* species, which likely form via residual H on the surface from the prereduction step. The CO_3_^2–^-related bands are not observed under SS cofeed conditions (Figure 3). Under SS conditions, where both H_2_ and CO_2_ are present, initial H_2_ activation favors adsorption of CO_2_ predominantly as HCOO* species rather than CO_3_^2–^*. During the conversion step (Figure 8B) of RCC, the m-CO_3_^2–^ and HCOO* bands decrease in intensity, while new CH_3_O* bands appear. These trends suggest that m-CO_3_^2–^* species could be initially hydrogenated to HCOO*, which are subsequently hydrogenated to CH_3_O* species.

**Figure 8:**
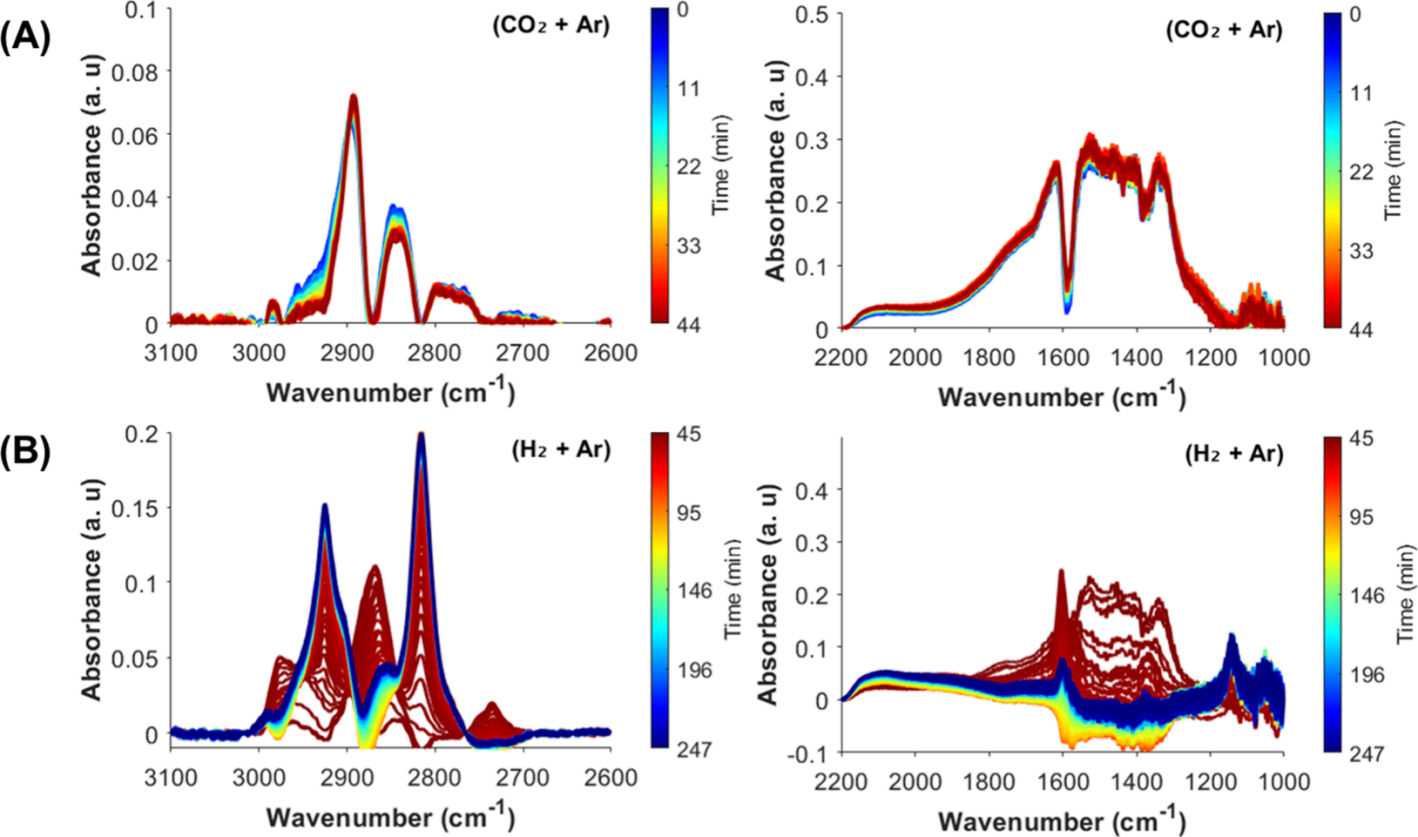
*In situ* DRIFTS under RCC conditions over the ZZO catalyst. (A) During the capture step at atmospheric pressure and 300 °C under 10% CO_2_/Ar. (B) During the conversion step at 6 bar and 300 °C in 60% H_2_/Ar. Reaction carried out at 6 bar and 300 °C.

Figure 9 presents the initial rates of MeOH, CO, and CH_4_ formation obtained from one RCC cycle in the fixed-bed reactor at different temperatures and H_2_ concentrations. Under RCC conditions, during the CO_2_ capture step, the surface becomes saturated by CO_2_-related adsorbed species. Thus, under RCC, only the H_2_ partial pressure could influence the initial rate. For all the species—MeOH, CO, and CH_4_—a linear dependence on H_2_ pressure can be observed. With decreasing H_2_ concentration, the initial rate of formation of all products decreases. This agrees with the first-order H_2_ dependence observed during SS cofeed fixed-bed kinetic measurements (vide supra). However, it is noteworthy that CH_4_ was not detected under the cofeed SS conditions. Interestingly, CH_4_ was predominantly formed at 300 °C and high H_2_ concentrations under RCC conditions using the CS. For the first 200 min, CH_4_ was continuously formed, reaching its maximum production at around 350 min (i.e., no more CH_4_ was detected in the effluent of the reactor by GC after this time). In the case of CO and MeOH, at 300 °C and 100% H_2_, product evolution is observed in two regimes. After an initial production, there is a plateau, between minutes 50–200, before product formation resumes. For a H_2_ concentration of 70%, this behavior was not observed and CH_4_ productivity significantly decreased to only 70 μmol/g, with the reaction being completed after ∼50 min. From these results (Figure 9F), we observe that CH_4_ exhibits a higher dependence on H_2_ partial pressure than does MeOH or CO.

**Figure 9:**
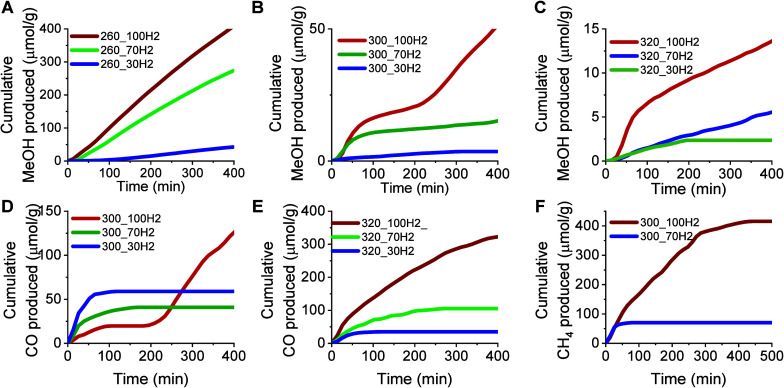
Initial rates of MeOH (A–C), CO (D, E), and CH_4_ (F) formation obtained from one RCC cycle at 260 °C (A), 300 °C (B, D, F), and 320 °C (C, E) at 6 bar under different H_2_ concentrations over ZZO + 10NaNO_3_/Mg_3_AlO_*x*_.

Figure 10 presents the *in situ* DRIFTS spectra under RCC conditions over the CS, ZZO + 10NaNO_3_/Mg_3_AlO*_x_*. During the CO_2_ capture step, an intense band appears at 1601 cm^–1^, attributed to adsorbed m-CO_3_^2–^* species (vide supra). In contrast to the SS cofeed conditions, no HCOO* species were detected during the capture phase (Figure 3). At 260 °C, upon switching the feed to H_2_ and increasing the pressure to 6 bar, bands associated with HCOO*—at 2736, 2875, and 2967 cm^–1^—and CH_3_O*—at 2817 and 2928 cm^–1^—increase in intensity over time. Since no gas-phase CO_2_ is present during the conversion step, these C-containing intermediates originate from the hydrogenation of surface-bound CO_3_^2–^* species. At 300 °C (Figure S3), an additional band at 3016 cm^–1^ appears, indicating CH_4_ formation at this temperature. At lower temperatures (e.g., 260 °C), only MeOH-related bands are observed, consistent with fixed-bed RCC results, where CH_4_ is only produced at 300 °C and high H_2_ partial pressure.

**Figure 10:**
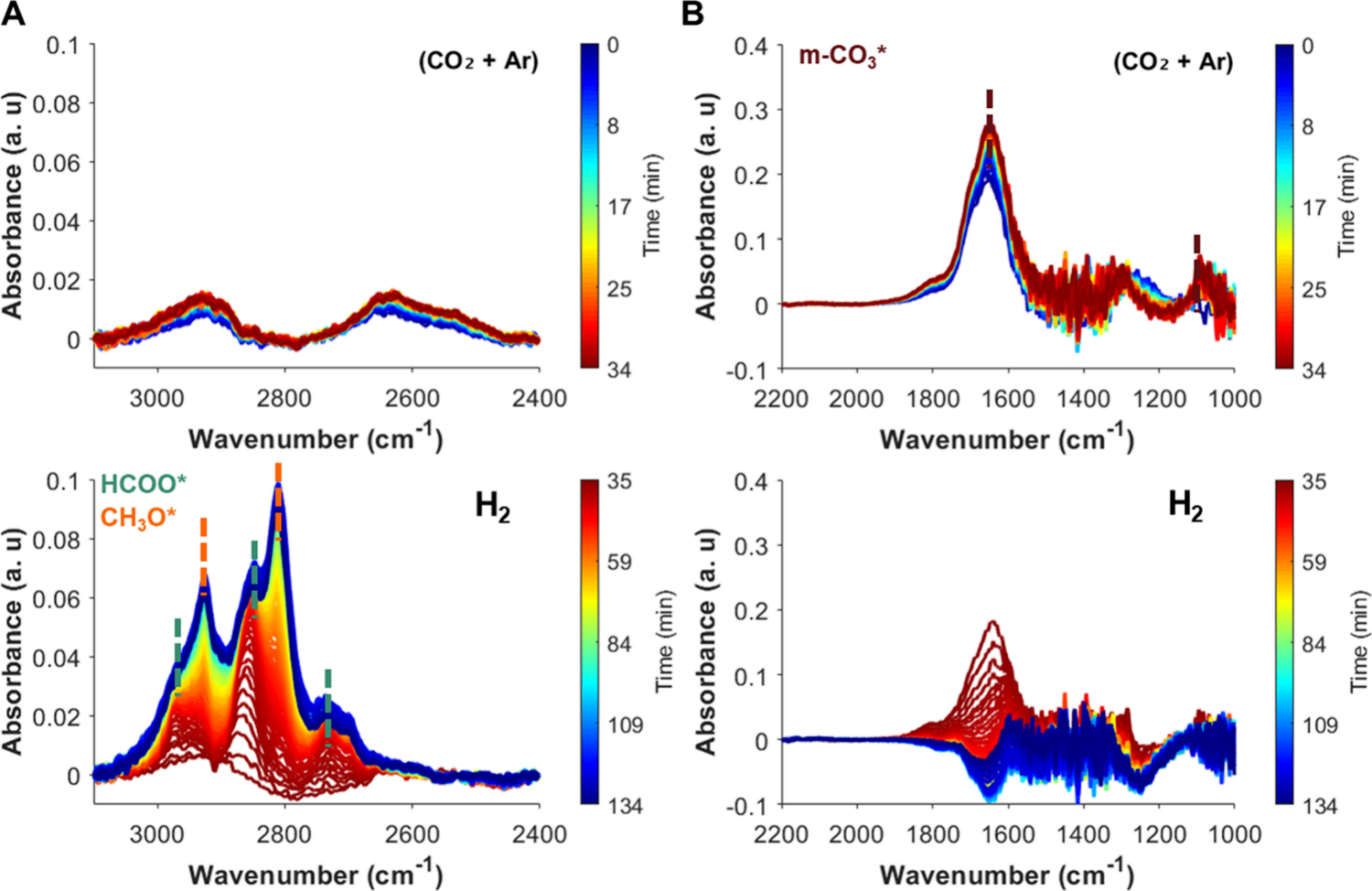
*In situ* DRIFTS under RCC conditions over ZZO + 10NaNO_3_/Mg_3_AlO*_x_* catalyst. (A) During the capture step at atm pressure and 260 °C under 10% CO_2_/Ar. (B) During the conversion step at 6 bar and 260 °C in H_2_/Ar.

To understand the CH_4_ formation mechanism further, additional fixed-bed and *in situ* DRIFTS RCC experiments were performed over the catalyst-free, NaNO_3_/Mg_3_AlO*_x_* sorbent alone. A variation of the standard RCC sequence was applied: after CO_2_ capture, a CO_2_ + H_2_ mixture was introduced instead of H_2_ alone. Under this condition, both CO and CH_4_ formation were observed—unlike in the standard cofeed SS case where no CH_4_ was detected. However, this occurred only at H_2_ concentrations greater than 75%. Following this experimental sequence, approximate reaction orders for CH_4_ and CO formation with respect to CO_2_ and H_2_ over NaNO_3_/Mg_3_AlO*_x_* were estimated (Figure 11A,B). CH_4_ exhibits a strongly negative order in CO_2_ (−1.3) and a highly positive order in H_2_ (+3), showing that CH_4_ formation proceeds from a surface-saturated carbon intermediate and is highly H_2_-dependent. On the other hand, CO exhibits a zero order dependence on CO_2_ and a first order dependence on H_2_, suggesting different reaction pathways for these two products. This was also observed by *in situ* DRIFTS (Figure 11C,D), where during the CO_2_ capture step, only the m-CO_3_^2–^* is observed, then during the H_2_ conversion step, the CH_4_ band at 3016 cm^–1^ appeared while the m-CO_3_^2–^* band decreased in intensity. Once CO_2_ is reintroduced, CO bands at 2111 and 2153 cm^–1^ appeared. Thus, the kinetic and spectroscopic evidence suggests that CH_4_ originates from hydrogenation of the m-CO_3_^2–^* reservoir, whereas CO formation likely proceeds via CO_2_ redox mechanism on Ov or through HCOO/COOH decomposition.

**Figure 11:**
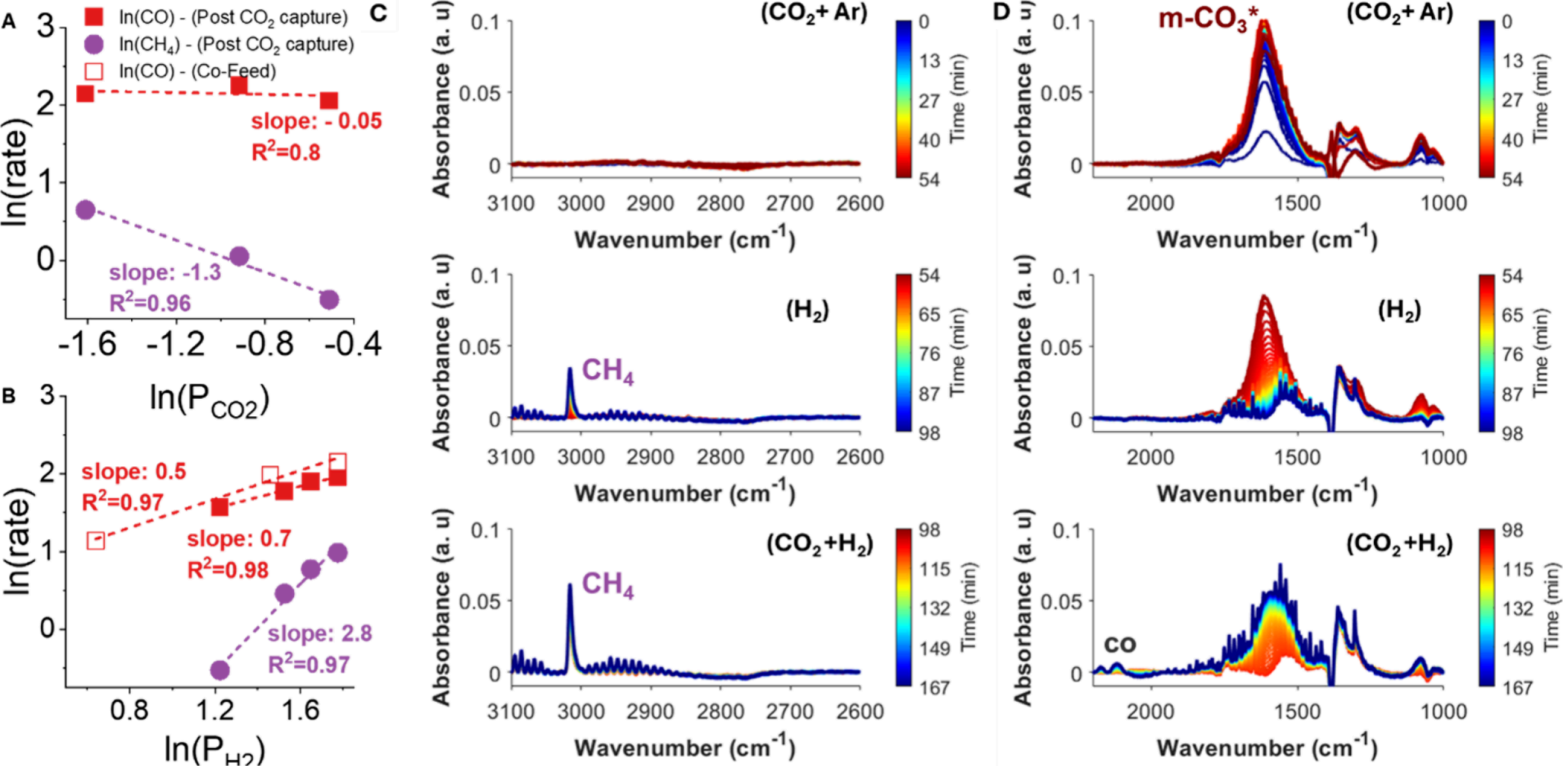
CO and CH_4_ reaction orders over NaNO_3_/Mg_3_AlO*_x_* for (A) CO_2_ and (B) H_2_ and *in situ* DRIFTS spectra of RCC to cofeed transition experiment over NaNO_3_/Mg_3_AlO*_x_* between (C) 3100–2600 cm^–1^ and (D) 2100–1000 cm^–1^.

Additional SS cofeed SSITKA-DRIFTS experiments were performed where only the H_2_ feed step was performed after the isotopic switch (Figure 12) using the catalyst-free sorbent. During the isotopic switch, the dominant m-CO_3_^2–^ band shifted from 1601 to 1536 cm^–1^, yet a residual feature near 1601 cm^–1^ persisted at the end of the ^13^CO_2_ switch, indicating incomplete exchange of the m-CO_3_^2–^ species, evidence of a slowly exchanging carbonate reservoir (Figure 12C). In the CO region, rovibrational ^12^CO features converted to ^13^CO, and a sharp band at ∼2077 cm^–1^ shifted to ∼2037 cm^–1^; because this band is also observed over ZZO alone, a tentative assignment is linearly bound CO on oxygen-deficient oxide sites common to both materials, though alternative carbonyl environments cannot be ruled out (Figure 12B). Although the same spectroscopic features are observed over the catalyst ZZO and the sorbent, NaNO_3_/Mg_3_AlO_*x*_, alone, different kinetics are observed. Over ZZO, CO has a first order dependence on CO_2_ and is zero order with respect to H_2_, while for the sorbent, NaNO_3_/Mg_3_AlO_*x*_, CO has a zero order dependence on CO_2_ and is first order with respect to H_2_. These differences likely arise from the intrinsic properties of the two materials: the sorbent exhibits higher CO_2_ affinity (CO_3_^2–^-rich surface) but limited H_2_ activation capacity, whereas ZZO has relatively low CO_2_ uptake but stronger H_2_ activation, resulting in a H-rich surface. The measured reaction orders therefore reflect these contrasting adsorption and activation characteristics.

**Figure 12:**
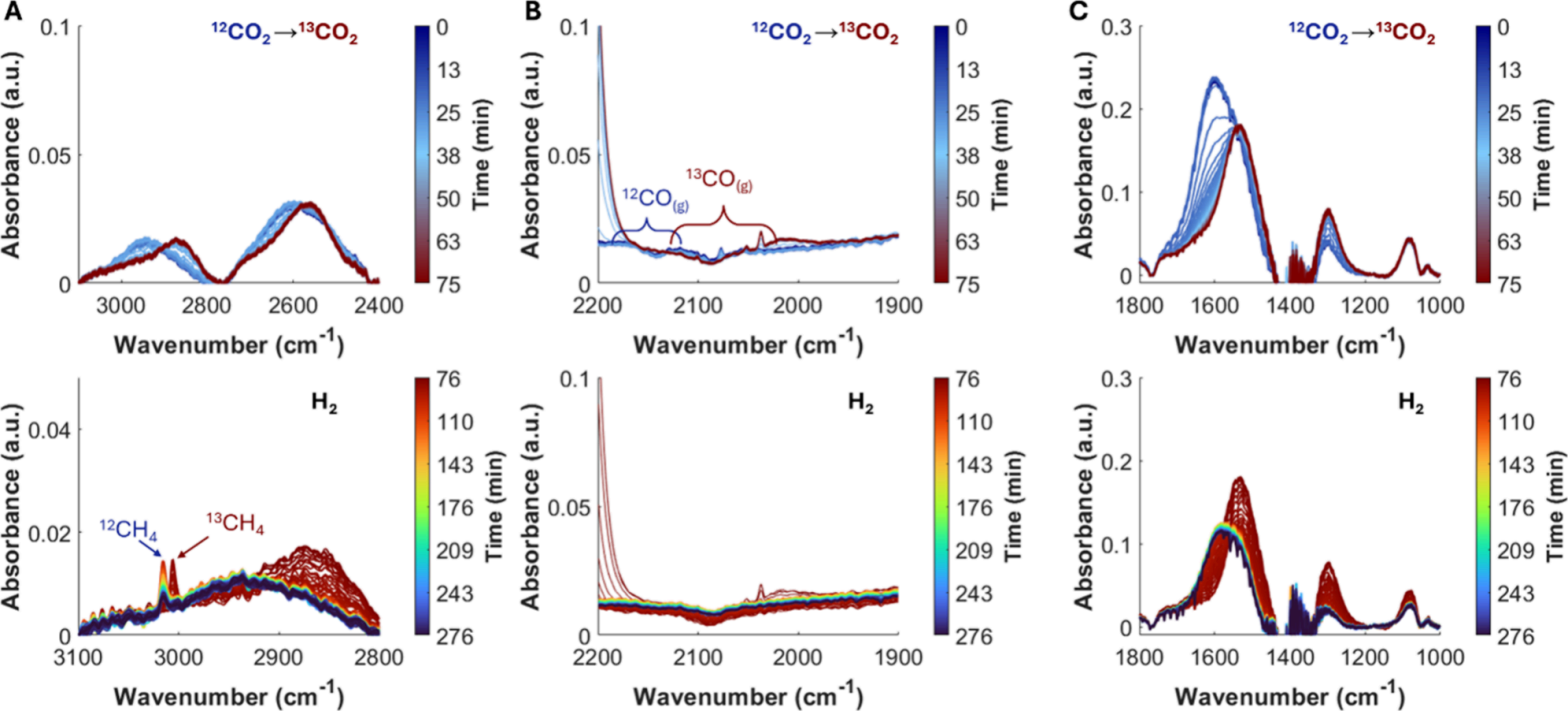
Time evolution of *in situ* DRIFTS spectra during the ^12^CO_2_ → ^13^CO_2_ isotopic switch (top panels) followed by H_2_-only feed (bottom panel) in the regions (A) 2400–3100 cm^–1^, (B) 1900–2200 cm^–1^, and (C) 1000–1800 cm^–1^ over 10NaNO_3_/Mg_3_AlO_*x*_.

In the spectral range between 2400–3100 cm^–1^ (Figure 12A) during the isotopic switch, broad bands at 2600 and 2900 cm^–1^ shift to lower wavenumbers. These are low-intensity, broad features that can be assigned to combination of carbonate modes, as their behavior mirrors that of the m-CO_3_^2–^ bands at 1601 and 1536 cm^–1^. Once the CO_2_ feed stops and only H_2_ is introduced, sharp peaks at 3005 and 3016 cm^–1^ appear, which correspond to ^13^CH_4_ and ^12^CH_4_, respectively. Interestingly, the 3005 cm^–1^ band disappears shortly before the 3016 cm^–1^ band appears.^14^ The sequential appearance of ^13^CH_4_ followed by ^12^CH_4_ (opposite of what would intuitively be expected) indicates that CH_4_ formation originates from CO_3_^2–^ species, with the initially formed ^13^CH_4_ reflecting the newly adsorbed CO_2_ and the delayed ^12^CH_4_ arising from the residual m-CO_3_ reservoir that persisted through the isotopic switch. DFT and molecular dynamics simulations (MDS) simulations examining direct MgCO_3_ hydrogenation pathways have shown that CH_4_ formation is favored via direct hydrogenation of surface-bound *CO_3_^2–^, with HCOO species as intermediates.^49^ However, in this study HCOO peaks over the NaNO_3_/Mg_3_AlO_*x*_ were not observed by DRIFTS, likely because their characteristic peaks overlap with the broad m-CO_3_^2–^ band at 1601 cm^–1^. Additional fixed-bed experiments were performed by exposing commercial MgCO_3_ (Millipore-Sigma) to 6 bar H_2_ at 300 °C, which resulted in CH_4_ formation but no detectable CO formation (Figure S7), consistent with a CO_3_-to-CH_4_ pathway.

A similar sequence was performed over the ZZO + 10NaNO_3_/Mg_3_AlO*_x_* CS with a ^13^CO_2_ capture step, followed by H_2_ feed at 6 bar, and then ^12^CO_2_ + H_2_ cofeed, and finally ^12^CO_2_ was removed from the feed by flowing only H_2_ (Figure S8). During the capture step, m-^13^CO_3_^2–^ bands were detected but no HCOO or CH_3_O* bands appeared. When H_2_ was introduced, a ^13^CH_4_ peak appeared together with ^13^HCOO and ^13^CH_3_O* bands, in contrast to the sorbent-only case, where CH_4_ was the only peak observed (Figures 11 and 12). This suggests that in the catalytic sorbent, some carbonate species remain in the sorbent domain and hydrogenate directly to CH_4_ under H_2_-rich conditions, while others migrate to the ZZO domain where they enter the HCOO*/CH_3_O* pathway leading to MeOH. Introduction of ^12^CO_2_ + H_2_ resulted in the growth of m-^12^CO_3_^2–^ and corresponding ^12^HCOO/CH_3_O* features, and the subsequent H_2_-only step produced a small ^12^CH_4_ peak alongside rising ^12^HCOO and CH_3_O* bands.

## Discussion

Our previous reports established that RCC performance can sometimes not be predicted from steady-state cofeed catalytic activity over the CS.^20^ Under SS cofeed conditions, MeOH and CO were the only products observed, but under RCC conditions CH_4_ emerged as the main product. These observations motivated the current work, where we explore the mechanistic origins of this distinction by combining kinetic analysis, *in situ* DRIFTS and isotopic switching experiments. The results presented here show that during the CO_2_ capture step, a carbonate-rich surface is established, primarily on the sorbent domain. Upon switching to H_2_, these carbonates follow two competing routes: direct hydrogenation on the sorbent domain, producing CH_4_, or migration of surface carbonates to the ZZO catalyst domain, where they are converted to MeOH through HCOO*/CH_3_O* intermediates. CH_4_ formation dominates under H_2_-rich conditions at 300 °C, while the appearance of MeOH could be linked to carbonate transfer to ZZO, which may explain the observed plateau in fixed bed experiments in MeOH productivity (Figure 9) at 300 °C. Moreover, our earlier study on CS configurations showed that a two-bed arrangement produced a product distribution resembling the sorbent alone, dominated by CH_4_, whereas decreasing the distance between catalyst and sorbent led to higher MeOH selectivity.^20^ This earlier observation is consistent with shorter diffusion paths facilitating CO_3_^2–^ mobility to the ZZO domain, thereby promoting MeOH formation. Under steady-state cofeed conditions, this dual reactivity is not observed, as CH_4_ is absent, highlighting that RCC enables pathways dependent on carbonate buildup and hydrogenation at high H_2_ partial pressures.

Fixed-bed RCC experiments further demonstrate that MeOH productivity decreases with decreasing H_2_ concentration (Figure 9). At low H_2_ concentration, carbonate species cannot be hydrogenated and are also resistant to desorption, since CO_3_^2–^ decomposition in inert gas occurs only at higher temperatures (∼550 °C, Figure S9). These findings emphasize that H_2_-rich conditions—rarely examined in conventional SS cofeed CO_2_ hydrogenation studies—are critical for determining the product distribution during RCC. Therefore, the development of catalytic sorbents for other RCC chemistries must explicitly evaluate carbonate reactivity under H_2_-rich conditions, where their hydrogenation and migration govern the product distribution. Such evaluation allows identification of whether carbonates are likely to hydrogenate directly, remain inert, or migrate to the catalyst where the intended intermediates (e.g., formate/methoxy for MeOH) can form. Incorporating this screening step into catalytic sorbent design is critical to avoid conditions that shift the system toward undesired CH_4_ production. In addition, the proximity between catalyst and sorbent domains should be carefully optimized, as the extent of carbonate transfer between these domains strongly influences whether CH_4_ or MeOH becomes the dominant product.

## Conclusions

In summary, the kinetics and mechanisms of CO_2_ hydrogenation over a ZnZrO_2_ catalyst and a ZnZrO_2_ + 10NaNO_3_/Mg_3_AlO*_x_* catalytic sorbent were investigated by fixed-bed kinetic measurements, *in situ* DRIFTS, and SSITKA-DRIFTS. CO_2_ adsorption over the 10NaNO_3_/Mg_3_AlO*_x_* sorbent was found to form Na_2_Mg­(CO_3_)_2_, as confirmed by XRD, consistent with mechanisms reported in the literature for NaNO_3_ molten-salt-promoted MgO sorbents. For the ZnZrO_2_ catalyst, MeOH formation proceeded via stepwise hydrogenation of HCOO to CH_3_O, while CO formation was attributed to CO_2_ dissociation at oxygen vacancies, as evidenced by the isotopic response of a sharp CO band at ∼2077 cm^–1^ and measured reaction orders. However, we cannot rule out a contribution from HCOO/COOH decomposition, since prior DFT studies have identified this pathway as energetically favorable. Thus, both mechanisms may contribute to the formation of CO under the conditions examined.

For the catalytic sorbent, ZnZrO_2_ + NaNO_3_/Mg_3_AlO_*x*_, distinct mechanistic features were observed. DRIFTS and isotopic studies indicated that CO_3_^2–^ species can spill over from the sorbent to the ZnZrO_2_ domain, where they undergo hydrogenation to MeOH through HCOO and CH_3_O intermediates. Under steady-state conditions, MeOH and CO were the dominant products, while CH_4_ was not detected.

In contrast, under RCC operation, the CO_2_ capture step formed a carbonate-rich surface, primarily on the sorbent domain. Upon switching to H_2_, these carbonates followed two competing routes: (i) direct hydrogenation within the sorbent, producing CH_4_, or (ii) migration to the ZnZrO_2_ catalyst domain, likely through the molten NaNO_3_, where they were converted to MeOH through HCOO and CH_3_O intermediates. CH_4_ formation dominated under H_2_-rich conditions at 300 °C, consistent with a high H_2_ reaction order measured on precarbonated material. On the contrary, MeOH formation was linked to carbonate transfer to ZnZrO_2_. These findings highlight that RCC introduces pathways not active under steady-state operation, since they depend on the buildup of carbonate and its hydrogenation under high H_2_ partial pressures, with the product distribution governed by the balance between direct carbonate hydrogenation and carbonate migration to the ZZO domain.

Despite these useful new insights, our study also has several limitations. The CO formation mechanism, and in particular the precise assignment of the carbonyl bands observed in the 2000–2100 cm^–1^ region, requires further validation through dedicated CO adsorption experiments and DFT calculations. Additionally, the use of a high-dead-volume DRIFTS cell limits the ability to quantitatively probe the mobility of carbonate species and their competing rates of hydrogenation versus spill over to the ZnZrO_2_ domain. Future studies using operando fixed-bed spectroscopy or low-dead-volume IR cells, combined with isotopic labeling, would provide more definitive insight into the dynamic interplay between CO_3_^2–^ hydrogenation to CH_4_ and CO_3_^2–^ migration to the catalyst for methanol synthesis.

## Supplementary Material



## Notes

X-ray diffraction patterns, *in situ* DRIFT spectra, mass spectrometry data, methane production rates and CO_2_ TPD results (PDF)
