# Supplementary material for: Reaction Pathways over ZnZrO2‑Based Catalysts and Catalytic Sorbents
Source: ACS Catal. 2025 Dec 31;16(2):1249–63. doi: 10.1021/acscatal.5c06895 (PMC12813983; doi:10.1021/acscatal.5c06895)
Supplement: Supplementary file 1 [file cs5c06895_si_001.pdf]

**Supporting Information for:**

# Reaction Pathways over ZnZrO2-based Catalysts and Catalytic Sorbents

Laura Proaño, Jordan Wielang, Christopher W. Jones*

School of Chemical & Biomolecular Engineering, Georgia Institute of Technology, Atlanta, GA 30332 (United States)

*Corresponding author: cjones@chbe.gatech.edu

Number of pages: 7

Number of figures:10

Number of tables: 0

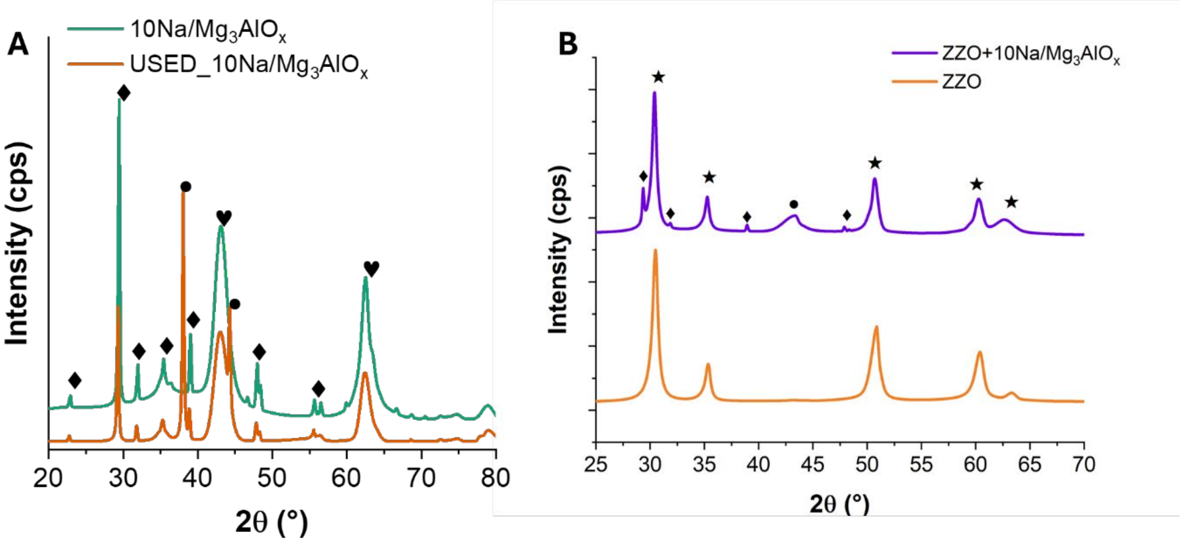


**Figure S1**. A) Extended XRD pattern of fresh and $CO_2$ exposed $10NaNO_3/Mg_3AlOx$ and (B) XRD pattern of fresh $ZnZrO_2$ catalyst and $ZnZrO_2$+$NaNO_3/Mg_3AlO_x$ after calcination(♦) $NaNO_3$ (●) MgO (♥) $Na_2MgCO_3$ (★)t-$ZrO_2$

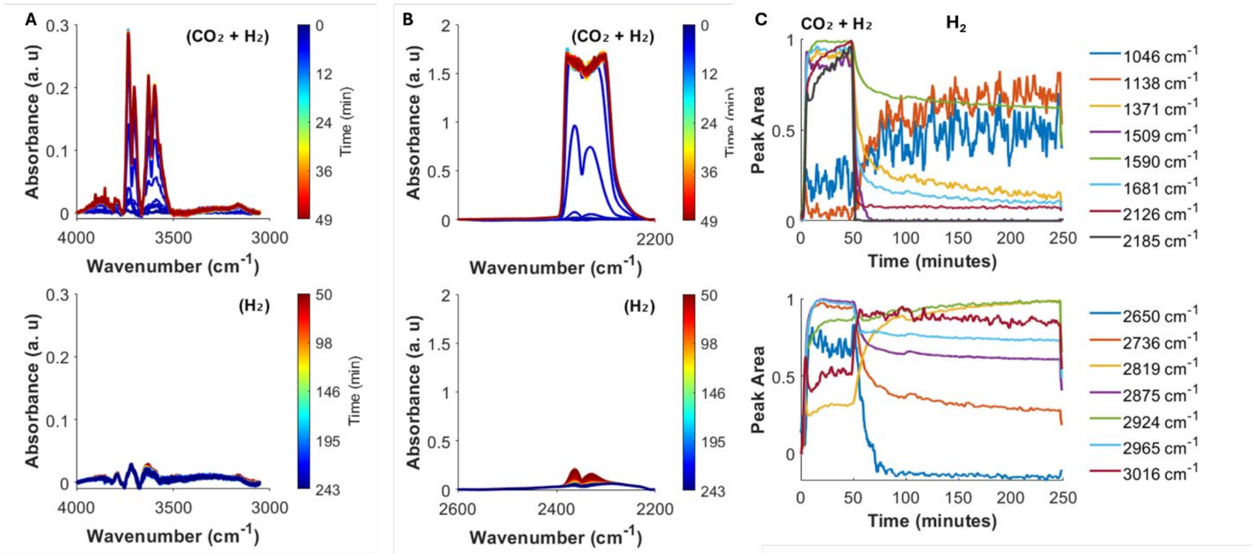


**Figure S2**. *In situ* DRIFTS under co-feed steady-state conditions over ZZO (A) Spectral region 2200–2600 $cm^{-1}$ and (B) region 3000–4000 $cm^{-1}$. Time evolution of spectra under SS co-feed conditions (10% $CO_2$/30% $H_2$/Ar, top panel) and after switching the feed to $H_2$ (bottom panel). (C) Evolution of normalized peak intensities for selected IR bands.

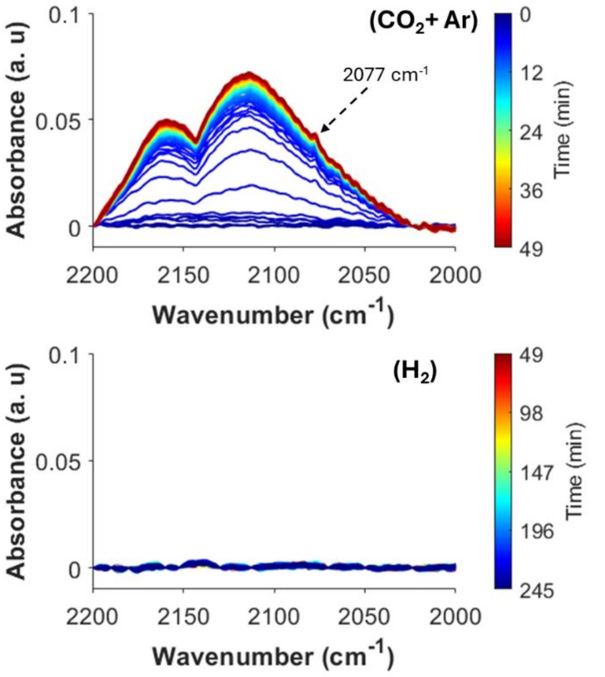


**Figure S3**. *In situ* DRIFTS under co-feed steady-state conditions over ZZO (A) Spectral region 2000–2200 cm-1. Time evolution of spectra under SS co-feed conditions (10% $CO_2$/30% $H_2$/Ar, top panel) and after switching the feed to $H_2$ (bottom panel).

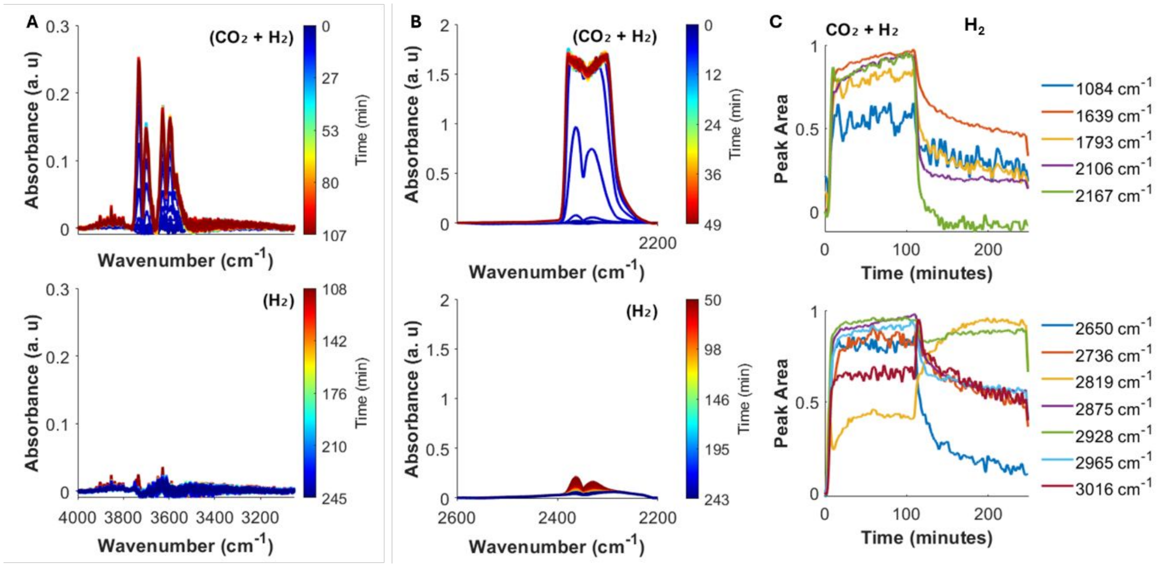


**Figure S4**. *In situ* DRIFTS under co-feed steady-state conditions over ZZO+10Na/$Mg_3AlO_x$ (A) Spectral region 2200–2600 $cm^{-1}$ and (B) region 3000–4000 $cm^{-1}$. Time evolution of spectra under SS co-feed conditions (10% $CO_2$/30% $H_2$/Ar, top panel) and after switching the feed to $H_2$ (bottom panel). (C) Evolution of normalized peak intensities for selected IR bands.

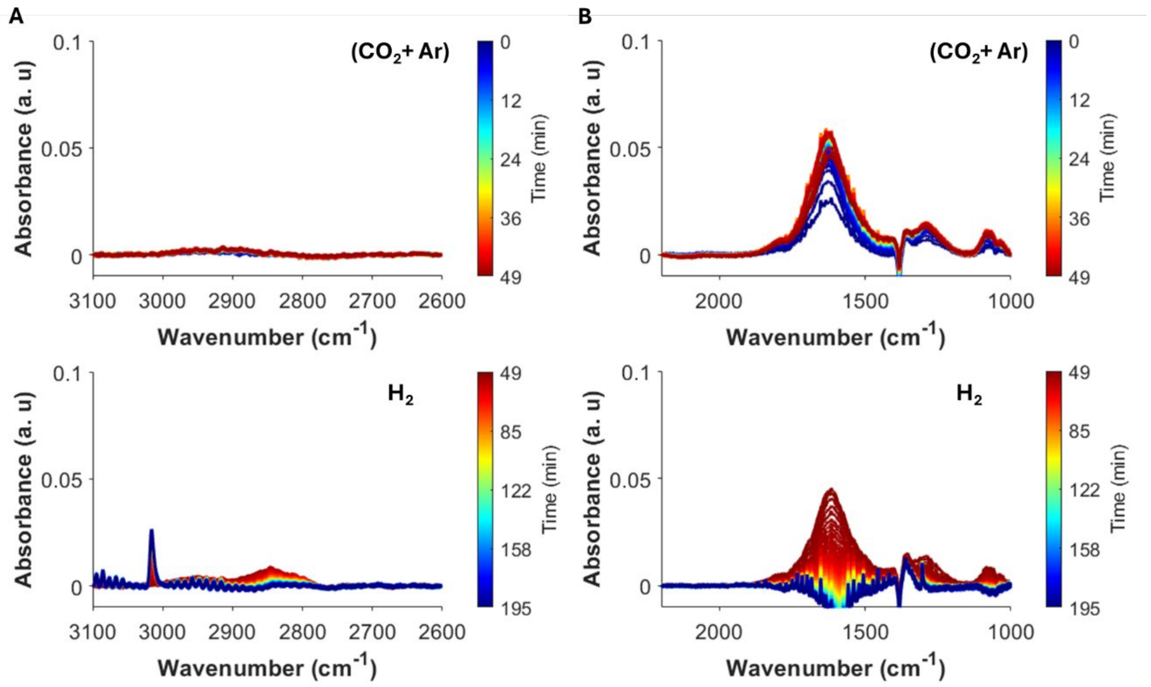


**Figure S5**. *in situ* DRIFTS under RCC conditions over ZZO+10Na/$Mg_3AlOx$ catalyst (A) during the capture step at atmospheric pressure and 300 °C under 10%$CO_2$/Ar and (B) during the conversion step at 6 bar and 260 °C in $H_2$.

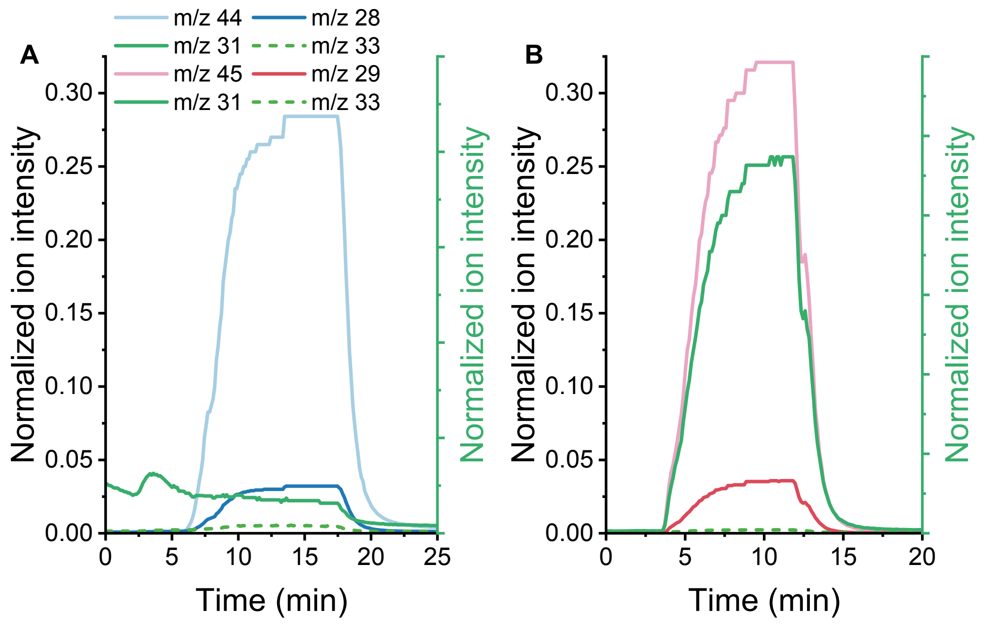


**Figure S6.** Intensity of characteristic m/z signals detected at the outlet of the DRIFTS cell during blank runs with (A) $^{12}CO2$ and (B) $^{13}CO2$.

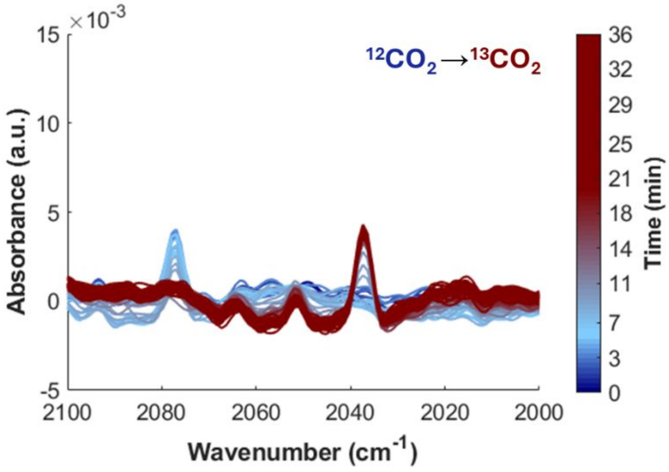


**Figure S7.** Evolution of *in situ* DRIFTS spectra in the region 2000–2100 cm$^{-1}$ cell during $^{12}CO_2$ → $^{13}CO_2$ switching under co-feed steady-state conditions over ZZO.

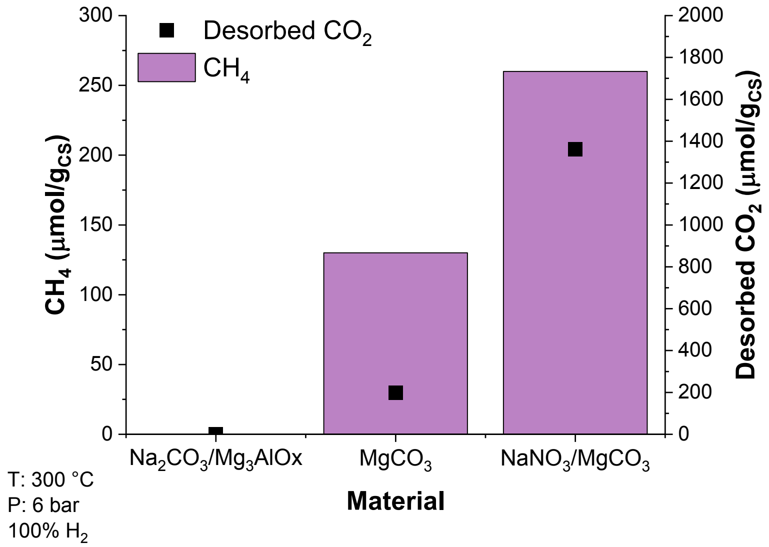


**Figure S8.** $CH_4$ and $CO_2$ desorption measured during exposure of $Na_2CO_3/Mg_3AlO_x$, $MgCO_3$, and $NaNO_3/MgCO_3$ to 6 bar $H_2$ at 300 °C

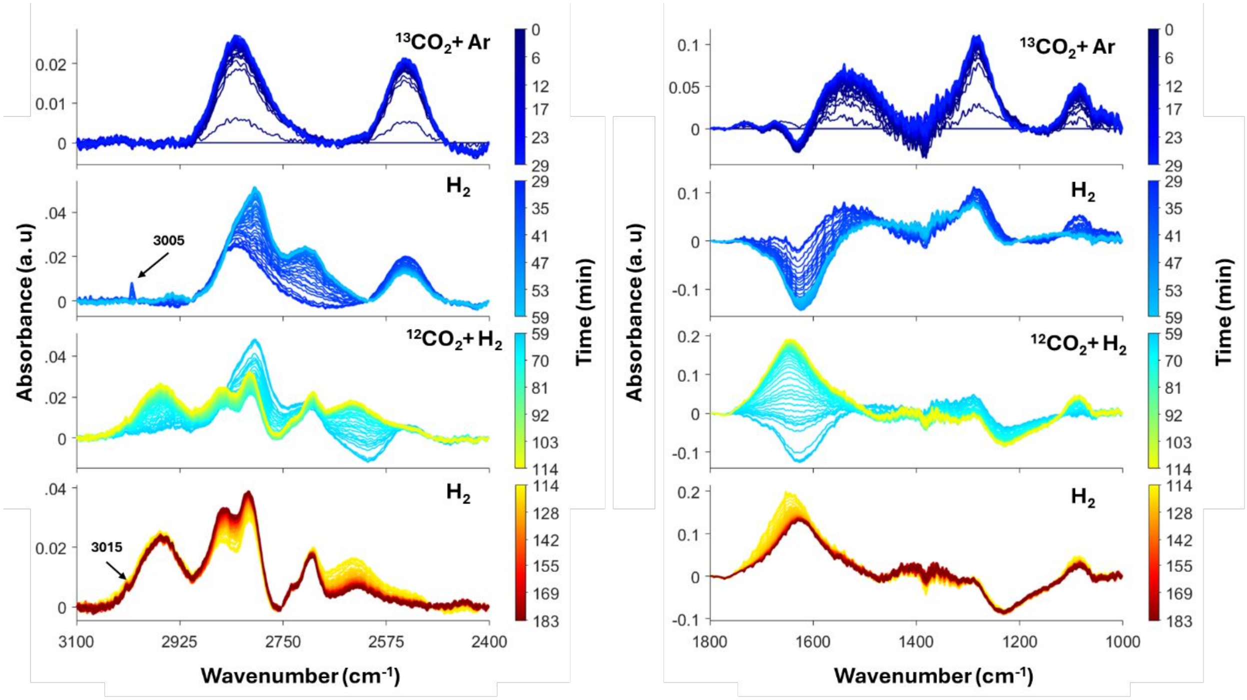


**Figure S9.** *In situ* DRIFTS spectra recorded over the ZZO+10$NaNO_3$/$Mg_3AlO_x$ catalytic sorbent during a sequence consisting of $^{13}CO_2$ capture, subsequent $H_2$ feed at 6 bar, introduction of $^{12}CO_2$+$H_2$ co-feed, and final $H_2$ feed step

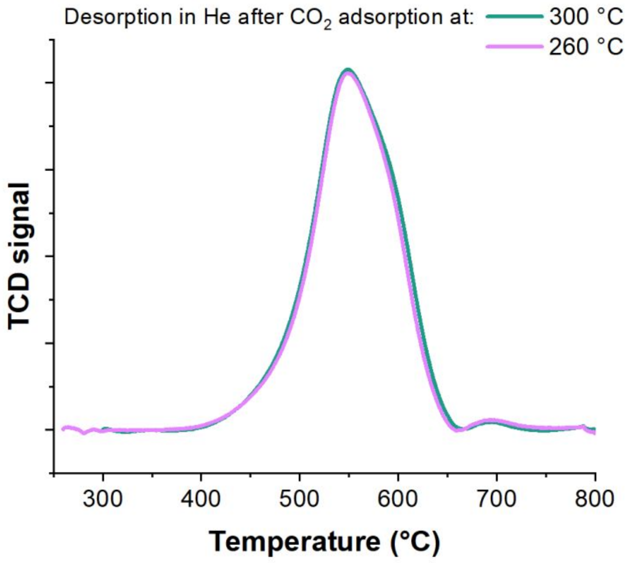


**Figure S10.** $CO_2$ TPD carried out after $CO_2$ adsorption on the 10$NaNO_3$/$Mg_3$AlOx sorbent at 260 and 300 °C
